# Small RNA profiling reveals regulation of Arabidopsis miR168 and heterochromatic siRNA415 in response to fungal elicitors

**DOI:** 10.1186/1471-2164-15-1083

**Published:** 2014-12-10

**Authors:** Patricia Baldrich, Klementina Kakar, Christelle Siré, Ana Beatriz Moreno, Angélique Berger, Meritxell García-Chapa, Juan José López-Moya, José Luis Riechmann, Blanca San Segundo

**Affiliations:** Centre for Research in Agricultural Genomics (CRAG) CSIC-IRTA-UAB-UB, Edifici CRAG, Bellaterra (Cerdanyola del Vallés), Campus UAB, 08193 Barcelona, Spain; Institució Catalana de Recerca i Estudis Avançats (ICREA), Barcelona, Spain

**Keywords:** Arabidopsis, Microarray analysis, microRNA, Fungal elicitors, hc-siRNA, miR168 sensor

## Abstract

**Background:**

Small RNAs (sRNAs), including small interfering RNAs (siRNAs) and microRNAs (miRNAs), have emerged as important regulators of eukaryotic gene expression. In plants, miRNAs play critical roles in development, nutrient homeostasis and abiotic stress responses. Accumulating evidence also reveals that sRNAs are involved in plant immunity. Most studies on pathogen-regulated sRNAs have been conducted in Arabidopsis plants infected with the bacterial pathogen *Pseudomonas syringae*, or treated with the flagelin-derived elicitor peptide flg22 from *P. syringae*. This work investigates sRNAs that are regulated by elicitors from the fungus *Fusarium oxysporum* in Arabidopsis.

**Results:**

Microarray analysis revealed alterations on the accumulation of a set of sRNAs in response to elicitor treatment, including miRNAs and small RNA sequences derived from massively parallel signature sequencing. Among the elicitor-regulated miRNAs was miR168 which regulates ARGONAUTE1, the core component of the RNA-induced silencing complex involved in miRNA functioning. Promoter analysis in transgenic Arabidopsis plants revealed transcriptional activation of *MIR168* by fungal elicitors. Furthermore, transgenic plants expressing a *GFP-miR168* sensor gene confirmed that the elicitor-induced miR168 is active. MiR823, targeting Chromomethylase3 (*CMT3*) involved in RNA-directed DNA methylation (RdDM) was also found to be regulated by fungal elicitors. In addition to known miRNAs, microarray analysis allowed the identification of an elicitor-inducible small RNA that was incorrectly annotated as a miRNA. Studies on Arabidopsis mutants impaired in small RNA biogenesis demonstrated that this sRNA, is a heterochromatic-siRNA (hc-siRNA) named as siRNA415. Hc-siRNAs are known to be involved in RNA-directed DNA methylation (RdDM). SiRNA415 is detected in several plant species.

**Conclusion:**

Results here presented support a transcriptional regulatory mechanism underlying *MIR168* expression. This finding highlights the importance of miRNA functioning in adaptive processes of Arabidopsis plants to fungal infection. The results of this study also lay a foundation for the involvement of RdDM processes through the activity of siRNA415 and miR823 in mediating regulation of immune responses in Arabidopsis plants.

**Electronic supplementary material:**

The online version of this article (doi:10.1186/1471-2164-15-1083) contains supplementary material, which is available to authorized users.

## Background

The genomes of higher eukaryotes encode small RNAs (sRNAs) that direct transcriptional and post-transcriptional gene silencing [1–3]. In plants, sRNAs can be categorized into two major classes including microRNAs (miRNAs) and small interfering RNAs (siRNAs) which are distinguished by their precursor molecules and different modes of biogenesis [4–6].

MiRNAs are derived from primary miRNA (pri-miRNA) transcripts that form an imperfect fold-back structure [7]. The pri-miRNA is then processed in a two-step pathway by a Dicer-like (DCL) ribonuclease, typically DCL1, to produce miRNA/miRNA* duplexes which are methylated and exported to the cytoplasm [7, 8]. Alternative pathways for miRNA biogenesis involving DCL3 or DCL4 have also been described [9, 10]. The miRNA is then selectively incorporated into the ARGONAUTE 1 (AGO1)-containing RNA-induced silencing complex (RISC) and thereby directs cleavage or translational inhibition of the target mRNA [11–13].

The second major class of sRNAs, includes siRNAs that are generated from long double-stranded RNAs (dsRNAs) resulting from the activity of RNA-dependent RNA polymerases (RDRs) that are sliced by DCL activities into siRNA duplexes [4, 14]. Thus, the most distinguishing feature of siRNA biogenesis is the requirement of RDR activity for generation of siRNA precursors, whereas miRNAs have single-stranded RNA precursors and do not require RDR activity for their biosynthesis. Plant siRNAs can be further categorized as heterochromatic siRNAs (hc-siRNAs; also referred to as repeat-associated siRNAs or ra-siRNAs), secondary siRNAs (including trans-acting siRNAs or ta-siRNAs), and natural antisense transcript-derived siRNAs (nat-siRNAs) [4]. Production and function of each class of siRNAs has very consistent requirements for specific members of the DCL, RDR and AGO gene families. Whereas DCL2 is mainly involved in the generation of nat-siRNAs [15, 16], DCL3 is responsible for the processing of RDR2-generated dsRNA and gives rise to 24-nt hc-siRNAs [14, 17]. DCL4 acts mainly in the biogenesis of ta-siRNAs in an RDR6-dependent manner [10, 18]. As for AGO proteins, the core components of the RISC complex, AGO1 primarily binds miRNAs and AGO4 binds hc-siRNAs [12, 19–21].

MiRNAs and siRNAs are further distinguished by their dependency on DNA-dependent RNA polymerases for their production. MiRNAs are typically transcribed by Pol II whereas hc-siRNA sequences are transcribed by Pol IV. The Pol IV transcripts serve as templates for RDR2 to generate dsRNAs that are processed by DCL3 into 24-nt hc-siRNAs and then loaded onto AGO4-containing complexes [22–24]. The AGO4-bound hc-siRNAs are recruited by nascent Pol V transcripts which then guide RNA-directed DNA methylation (RdDM). Along with this, Pol IV and Pol V have distinct roles in the RdDM pathway, Pol IV being required for transcription of precursor RNAs from heterochromatic loci, and Pol V transcripts being required for siRNA targeting to the RdDM-affected loci.

During the last years the number of plant miRNAs registered in miRBase (http://microrna.sanger.ac.uk) has dramatically increased, this expansion being largely a benefit of the adoption of next-generation high-throughput sequencing technology. Although the early criteria for miRNA annotation based on expression and biogenesis still provide a broadly accepted standard for miRNA annotation [25], additional criteria were proposed to strengthen plant miRNA annotations (i.e. identification of miRNA and miRNA* sequences and DCL dependency for miRNA accumulation) [26]. However, some released miRNAs might still be incorrectly annotated and not assessed with sufficient stringent criteria prior to their addition to the database.

Plant miRNAs are known to play important roles in a wide range of developmental processes [27, 28]. MiRNAs also regulate the miRNA pathway itself [29]. There is also increasing evidence that the modulation of miRNA levels plays an important role in reprogramming plant responses to abiotic stress, including drought, cold, salinity, and nutrient deficiency [30, 31]. In Arabidopsis, miR396 acts as a developmental regulator in the reprogramming of root cells during cyst nematode infection [32]. New insight into miRNA function was gained with the discovery that distinct miRNAs target genes involved in plant immune responses to pathogen infection [33–38].

Traditionally, studies on plant immunity focused on the transcriptional regulation of protein-coding genes. Along with this, host-encoded receptors recognize pathogen-associated molecular patterns (PAMPs, previously known as elicitors). This recognition elicits the PAMP-triggered immunity (PTI), or basal disease resistance, a process in which regulation of immune-response genes occurs through the coordinated regulation of hormone signals [39]. Some pathogens, in turn, deliver effector proteins into the host cell that interfere PTI functions and allow successful infection. As another layer of defence, plants have developed the ability to recognize such microbial effectors by additional receptors (resistance proteins, R) to activate the effector-triggered immunity (ETI). In this context, it is becoming apparent that small RNAs can modulate host gene expression in both PTI and ETI [33–38]. The important role of the Arabidopsis miR393 in antibacterial resistance is well documented [40]. Thus, infection with the bacterial pathogen *P. syringae* as well as treatment with the bacterial elicitor flagelin (flg22, a well-studied PAMP from *P. syringae* flagellin) induces miR393 accumulation which, in turn, silences the expression of the TIR1 (TRANSPORT INHIBITOR RESPONSE1) auxin receptor. Repression of auxin signaling then contributes to bacterial resistance [40]. MiRNAs that guide the cleavage of transcripts corresponding to *R* genes and trigger production of phased secondary siRNAs have been characterized in Solanaceae and Leguminosae species in relation to antiviral and antibacterial resistance [34, 37]. In rice, we recently described a miRNA, miR7695 that positively regulates resistance to infection by the rice blast fungus *Magnaporthe oryzae* [41]. Indeed, together with the knowledge of pathogen-responsive genes (i.e. plant antifungal genes), a better knowledge of sRNAs involved in plant immunity will contribute to delineate novel strategies to improve disease resistance in plants.

Most studies on pathogen-regulated sRNAs have been conducted in Arabidopsis plants infected with the bacterial pathogen *Pseudomonas syringae,* or treated with the flagelin-derived elicitor peptide flg22 from *P. syringae* [40]. However, less is known about sRNAs that are regulated during infection with fungal pathogens in plants. To fill this knowledge gap, we conducted a microarray-based search to identify Arabidopsis small RNAs whose expression is affected by treatment with fungal elicitors. Among the elicitor-regulated miRNAs was miR168, known to control AGO1 homeostasis. Both the precursor and mature miR168 were induced by elicitor treatment. Consistent with this, the *MIR168a* promoter is transcriptionally activated by fungal elicitors in transgenic Arabidopsis plants harboring the *promMIR168a::GFP* (green fluorescent protein) fusion gene. Using transgenic plants expressing a GFP-miR168* sensor construct, we further demonstrated that the elicitor-induced miR168 is active. Microarray analysis also revealed elicitor responsiveness of a small RNA currently annotated as miR415 in miRBase. The status of this sequence has been, however, questioned (http://microrna.sanger.ac.uk). Using Arabidopsis mutants impaired in small RNA biogenesis and function, we show that production of this particular small RNA depends on Pol IV, DCL3 and RDR2 activities. Based on these findings, we conclude that this small RNA is a hc-siRNA, and can no longer be considered a miRNA. Microarray experiments also revealed elicitor-responsiveness of miR823, this miRNA targeting the plant-specific methyltransferase involved in DNA methylation (Chromomethylase3, CMT3). These findings further support a role for hc-siRNAs, potentially acting in RdDM processes, in the response of Arabidopsis plants to fungal elicitors.

## Results

### Identification of small RNAs that are responsive to fungal elicitors in Arabidopsis

In this work, we examined alterations in the accumulation of Arabidopsis small RNAs in response to treatment with elicitors obtained from the fungus *Fusarium oxysporum* [42]. Towards this end, we used a customized microarray containing 2382 probes corresponding to 166 known miRNAs (i.e. small RNAs mapping at the 5p or 3p arm of the precursor structures annotated in miRBase), 553 candidate miRNAs selected from the literature [10, 43, 44] and 1096 small RNA sequences derived from massively parallel signature sequencing (MPSS) in Arabidopsis [45, 46]. Probes for known miRNAs from non-plant species (*Caenorhabditis elegans, Drosophila melanogaster*) were also included in the microrray. The complete list of probes represented in the microarray is presented in Additional file 1: Table S1.

Total RNA was isolated from Arabidopsis seedlings that had been treated with elicitors obtained from the fungus *F. oxysporum*, and mock-inoculated plants, at different times of treatment (from 5 min to 120 min of treatment). Three independent biological replicates were prepared for each time point and condition. Samples harvested at 5, 30, 60 and 120 min of elicitor treatment were used for microarray experiments. Elicitor-induced alterations in small RNA accumulation we identified by determining the ratio of the hybridization signal intensities between treated and control plants.

Among the small RNAs interrogated in the microarray, 15 miRNAs corresponding to 13 miRNA families, and 81 predicted miRNAs showed elicitor-responsiveness (up- or down-regulation) at one or more time points of elicitor treatment (p value ≤ 0.05; Table 1 and Additional file 1: Table S1). Most of these differentially expressed miRNAs showed a dynamic response to fungal elicitors. Only miR168 maintained a constant trend (up-regulation) in its response to fungal elicitors during the entire period of treatment.

**Table 1:** **Elicitor-responsiveness of known Arabidopsis miRNAs as determined by microarray analysis**

Name	Sequence	Direction of miRNA expression				Target gene	Biological function
		5 min	30 min	60 min	120 min		
**miR156a**	ugacagaagagagugagcac	-	-	-	-1,79	SPL10 TF (At1g27370) ^1^	Development
**miR156h**	ugacagaagaaagagagcac	-4,13	-2,74	1,41	-	SPL2 ( At5g43270) ^2^	
**miR164a**	uggagaagcagggcacgugca	-	1,16	-	-	CUC1/2 TF (At5g53950/At3g15170) ^2^	Development. Auxin signaling
**miR164c**	uggagaagcagggcacgugcg	-	9,5	-	-	NAC080 TF/NAC100 TF (At5g07680/At5g61430) ^3^	
**miR165a**	ucggaccaggcuucauccccc	1,31	-	-	-	PHABULOSA TF/ PHAVOLUTA ^TF^ (At2g34710/At1g30490) ^4^	Development
**miR166a**	ucggaccaggcuucauucccc	-1,29	-	-	-		
**miR168**	ucgcuuggugcaggucgggaa	2,88	7,57	2,32	2,1	Argonaute1 (AGO1) (At1g48410) ^1^	miRNA functioning. Abiotic stress
**miR169d**	ugagccaaggaugacuugccg	-	-	-	-2,32	ATHAP2B (At3g05690) ^5^	Development. Auxin signaling
**miR170**	ugauugagccgugucaauauc	-	-1,76	-	-	SCL TF ^6^	Development
**miR415**	aacagagcagaaacagaacau	-	5,25	-	-	questioned miRNA	
**miR418**	uaaugugaugaugaacugacc	-	-	-	6,63	questioned miRNA	
**miR823**	uggguggugaucauauaagau	-	10,66	-	-	Chromomethylase 3 (CMT3) (At1g69770) ^2^	Gene silencing
**miR833a-5p**	uguuuguuguacucggucuagu	4,55	3,1	2,83	-1,28	F-box containing protein (At1g77650) ^2^	
**miR842**	ucauggucagauccgucaucc	-	-	-	3,09	Jacalin lectin (At5g28520) ^7^	
**miR862-5p**	uccaauaggucgagcaugugc	-	-1,73	-	-	Unknown	

^1^ [47] ^2^ [17]; ^3^ [27]; ^4^ [29]; ^5^ [2]; ^6^ [13]; ^7^ [48]. SPL, Squamose promoter binding protein-like; CUC1/2 TF, cup-shaped cotyledon1/2 transcription factor; NAC (NAM, ATAF and CUC) transcription factor; SCL, Scarecrow-like (GRAS TF). -, no change in expression.

miRNAs whose expression varies in at least one time point of elicitor treatment are listed (for details on the entire set of miRNAs represented in the microarray, see Additional file 1: Table S1. The fold change (elicitor-treated *vs* non-treated plants) for each miRNA is shown. Three biological replicates and three technical replicates for each biological sample were analysed.

Elicitor-regulated miRNAs mainly target transcription factors involved in the control of developmental processes. For instance, miR156 is known to target *SQUAMOSA PROMOTER BINDING PROTEIN LIKE* (*SPL*) transcription factors. Two members of the miR156 family, miR156a and miR156h, were found to be sequentially regulated by elicitors (Table 1). Among the group of elicitor-regulated miRNAs were also miR165/166 targeting the PHABULOSA and PHAVOLUTA transcription factors, miR170 targeting SCARECROW-like (SCL), and miR172 targeting APETALA2 transcription factors. Moreover, several miRNAs that regulate auxin homeostasis were identified among the set of elicitor-responsive miRNAs. They were: miR167 targeting two Auxin Response Factors (ARF) genes (*ARF6* and ARF8), miR164 and miR169 (targeting *NAC1* and subunits A of the NF-Y transcription factor complex, respectively). This observation points to a possible miRNA-mediated regulation of auxin signaling by fungal elicitors.

Of interest, miR168 was found to be up-regulated by fungal elicitors at all the time points of elicitor treatment examined here. We also noticed a significant increase in miR823 accumulation (FC, 10.66) at 30 min of elicitor treatment. This particular miRNA has been reported to target transcripts of *Chromomethylase 3* (*CMT3*) encoding an enzyme involved in DNA methylation and gene silencing [10]. Microarray analysis also showed regulation by fungal elicitors of certain small RNA sequences whose status as a miRNA has been questioned (i.e. miR415 and miR418).

Next, the conservation of elicitor-responsive miRNAs among different plant species, both dicotyledonous and monocotyledonous species, was investigated. In addition to *Arabidopsis thaliana*, *Medicago truncatula*, *Glycine max*, *Populus trichocarpa*, and *Solanum tuberosum* are the dicot species with the highest number of miRNAs annotated in the miRBase registry. As for monocots, *Oryza sativa*, *Zea mays*, *Sorghum bicolour* and *Brachypodium distachyon* have the highest number of miRNAs annotated in miRBase. In this work, conserved miRNAs were designated as those having identical sequences with the miRNA sequences annotated in miRBase for all the above mentioned plant species. Based on the information available on miRBase, both conserved and non conserved miRNAs were identified among the subset of elicitor-responsive miRNAs (Table 2).

**Table 2:** **Conservation among different plant species of elicitor-responsive miRNAs**

Name	Dicot				Monocot			
	Mt	Gm	Pt	St	Os	Bd	Zm	Sb
**miR156a**	(+)	(+)	(+)	(+)	(+)	(+)	(+)	(+)
**miR156h**	(+)	(+)	(+)	(+)	(+)	(+)	(+)	(+)
**miR164a**	(+)	(+)	(+)	(+)	(+)	(+)	(+)	(+)
**miR164c**	(+)	(+)	(+)	(+)	(+)	(+)	(+)	(+)
**miR165a**	(-)	(-)	(-)	(-)	(-)	(-)	(-)	(-)
**miR166a**	(+)	(+)	(+)	(+)	(+)	(+)	(+)	(+)
**miR168**	(+)	(+)	(+)	(+)	(+)	(+)	(+)	(+)
**miR169d**	(+)	(+)	(+)	(+)	(+)	(+)	(+)	(+)
**miR170**	(-)	(-)	(-)	(-)	(-)	(-)	(-)	(-)
**miR415**	(-)	(-)	(-)	(-)	(+)	(-)	(-)	(-)
**miR418**	(-)	(-)	(-)	(-)	(+)	(-)	(-)	(-)
**miR823**	(-)	(-)	(-)	(-)	(-)	(-)	(-)	(-)
**miR833a-5p**	(-)	(-)	(-)	(-)	(-)	(-)	(-)	(-)
**miR842**	(-)	(-)	(-)	(-)	(-)	(-)	(-)	(-)
**miR862-5p**	(-)	(-)	(-)	(-)	(-)	(-)	(-)	(-)

Mt, *Medicago truncatula*, Gm, *Glycine max*, Pt, *Populus trichocarpa*, St, *Solanum tuberosum*, Os, *Oryza sativa*, Bd, *Brachypodium distachyon*, Zm, *Zea mays*, Sb, *Sorghum bicolor.* + and -, identical and nonconserved sequences, respectively).

The microarray analysis comparing control non treated and elicitor-treated tissues of Arabidopsis plants also allowed us to examine the accumulation level of small RNA sequences previously identified through MPSS [45, 46]. Up to 98 of those small RNA sequences showed alterations in their accumulation at one or another time point (Additional file 1: Table S1). As it was observed for miRNAs, the response of these small RNA sequences to elicitors was highly dynamic, their accumulation being up- and down-regulated during the period of elicitor treatment examined here. Together, microarray analysis revealed an altered expression of a subset of sRNAs, including miRNAs from Arabidopsis, in response to treatment with fungal elicitors, which might be indicative of their possible involvement in the plant response to fungal infection.

Based on the results obtained in microarray experiments, two small RNA sequences were selected for further analysis, namely miR168 and the small RNA sequence annotated as miR415 in miRBase. miR168 was considered of interest due to the important role that this particular miRNA plays in controlling the miRNA machinery, by regulating AGO1 homeostasis. Concerning miR415, the status of this sequence as a miRNA has been questioned. In this context, it was important to investigate whether the elicitor-regulated small RNA sequence named as miR415 in miRBase is a true miRNA from Arabidopsis, or not.

### *MIR168*is transcriptionally activated in response to fungal elicitors in Arabidopsis

**Figure 1:**
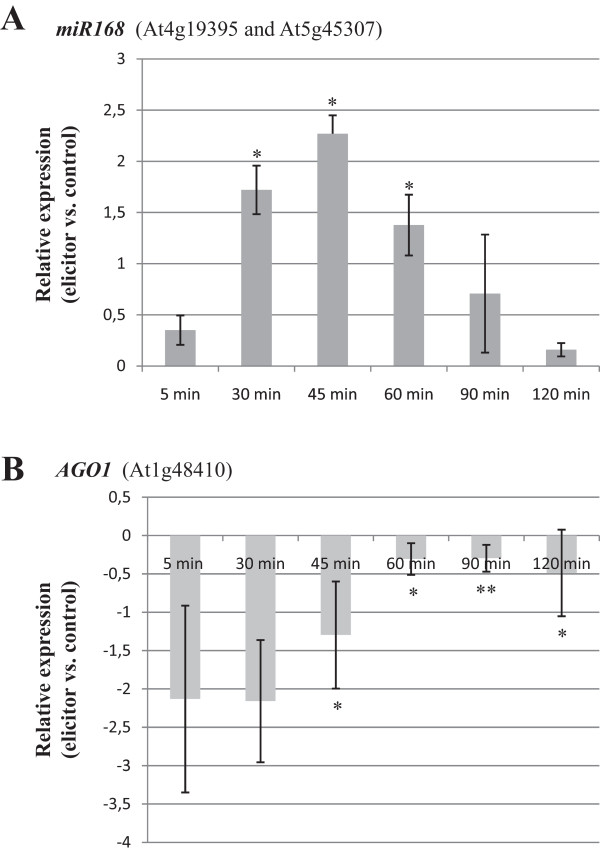
**Expression of miR168 and** ***AGO1*** **in Arabidopsis plants treated with elicitors from** ***F. oxysporum*** **. (A)** Stem-loop RT-qPCR analysis of miR168. RNAs were prepared from Arabidopsis plants treated with fungal elicitors for the indicated periods of time, and from control mock-inoculated plants (RNAs from samples harvested at 5, 30, 60 and 120 min were also used for microarray analysis). **(B)** qRT-PCR analysis of *AGO1* using *Ubiquitin10* (At5g65080) as the internal control. RNAs samples were the same as in (A). Results shown are from one of three independent experiments that gave similar results. Erro bars show the standard error. Asterisks indicate a significant difference between conditions (*, P ≤ 0.05 ; **; ≤0.01). c, control plants. e, elicitor-treated plants.

Microarray analysis revealed up-regulation of miR168 in response to elicitor treatment. This particular miRNA directs cleavage of *AGO1* mRNA, with AGO1 being the core component of the RISC complex involved in miRNA functioning [12]. Considering the important role of miR168 in controlling the miRNA machinery, it was of interest to explore the elicitor-responsiveness of both partners, miR168 and *AGO1*. Initially, stem-loop RT-qPCR analysis was used to examine mature miR168 accumulation in control and elicitor-treated plants at all time points of elicitor treatment (5, 30, 45, 60, 90 and 120 min). As it is shown in Figure 1A, a marked increase in miR168 accumulation could be observed as early as 5 min after the onset of elicitor treatment. miR168 accumulation remained at higher levels in elicitor-treated plants compared to control plants up to 90 min of elicitor treatment. Thus, results obtained by stem-loop RT-qPCR analysis were then consistent with those observed by microarray analysis, further supporting that miR168 is up-regulated by fungal elicitors. On the other hand, *AGO1* expression remained at lower level in elicitor treated tissues compared to control plants at all time points, although these differences gradually decreased during the period of elicitor treatment (Figure 1B).

Collectively, microarray and quantitative PCR analyses revealed that the mature miR168 accumulates in response to fungal elicitors. Moreover, the elicitor-induced up-regulation of miR168 might account for the observed reduction in *AGO1* transcripts in elicitor treated plants compared to control plants during the period of treatment here assayed.

**Figure 2:**
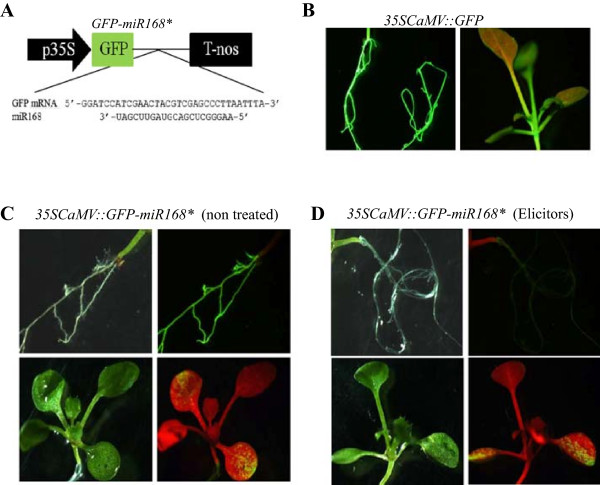
**MiR168 activity in Arabidopsis plants revealed by GFP fluorescence patterns tissues of** ***GFP*** -miR168* sensor plants. (A) Schematic representation of the miR168 sensor construct containing the *GFP* mRNA with a site complementary to miR168 (*GFP*-miR168*). **(B)** Plants constitutively expressing the *GFP* gene. Ten day**-**old plants were treated for 30 min with elicitors obtained from the fungus *F. oxysporum*. Water was used as mock control. Results obtained in elicitor-treated *GFP*-Arabidopsis plants are presented (similar patterns were observed in non treated *GFP*-Arabidopsis plants). GFP fluorescence images are shown. **(C)** Analysis of miR168 activity in control, non treated GFP-miR168* plants (*sde1* background). Bright-field (left) and GFP fluorescence (right) images are shown. **(D)** miR168 activity in elicitor-treated *GFP*-miR168* Arabidopsis plants. Bright-field (left) and GFP fluorescence (right) images are shown. No GFP fluorescence was evident in roots of non treated plants due to miR168 guided silencing.

Next, we investigated the *in vivo* activity of miR168 in tissues (leaves, roots) of control and elicitor-treated Arabidopsis plants. Despite the relevance of *MIR168* in controlling AGO1 homeostasis, little attention has been paid to *MIR168* expression or activity in tissues of Arabidopsis plants other than leaves. Towards this end, a sensor gene was obtained in which the miR168 target sequence was inserted into the 3’-UTR of the *GFP* reporter gene (GFP-miR168* gene, Figure 2A). Transgenic plants harboring the miR168 sensor gene were generated. MiR168 activity was inferred by monitoring GFP fluorescence in tissues of transgenic Arabidopsis plants (leaves, roots), and in response to treatment with fungal elicitors (the GFP-miR168* sensor is degraded in cells where miR168 is present). Transgenic plants expressing the *GFP* gene devoid of the miR168 target sequence were produced and used as controls. As expected, GFP fluorescence was visualized in roots and leaves of control *GFP*-Arabidopsis plants (Figure 2B).

**Figure 3:**
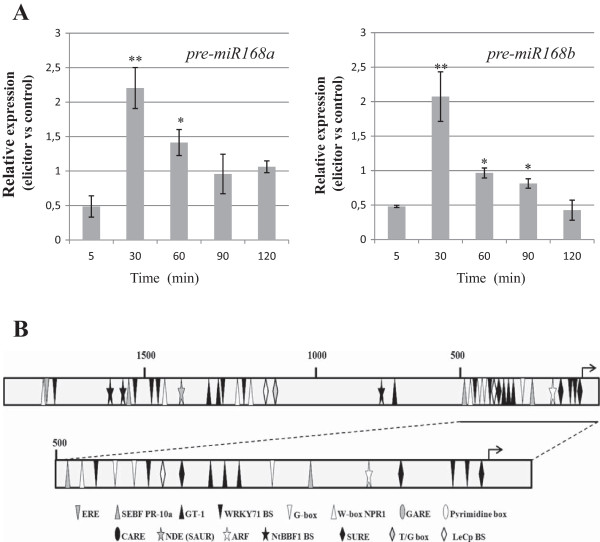
**Expression of miR168 precursors and structural features of the** ***MIR168*** **promoter. (A)** qRT-PCR analysis of pre-miR168a (left panel) and pre-miR168b (right panel) expression in response to elicitor treatment. The relative expression level in comparison to the corresponding non treated controls is given for each time point (elicitor *vs* control non treated plants). Error bars represent the mean ± SD of two biological replicates and three technical replicates for each biological replicate (*, P ≤ 0.05 ; **; ≤0.01). All values were normalized against *Ubiquitin*. **(B)** Structural features of the *MIR168a* promoter from Arabidopsis. The location of known *cis*-acting elements is shown (for details on *cis*-elements, see Additional file 1: Table S2).

Under control conditions, *GFP* expression was readily detectable in roots of GFP-mir168* plants whereas only some fluorescent spots were visualized in their leaves, mainly at the distal areas (Figure 2C). Then, we compared the GFP patterns in tissues of control and elicitor-treated GFP-miR168* Arabidopsis plants. Remarkably, treatment with fungal elicitors resulted in loss of GFP fluorescence in roots of *GFP*-miR168* plants suggesting miR168 activity in these tissues (Figure 2D, left panels). By contrast, differences in GFP fluorescence pattern between control and elicitor-treated leaves of *miR168-GFP* plants were not evident (Figure 2D, right panels). These findings support that elicitor treatment results in enhanced activity of miR168, mainly in roots of Arabidopsis plants. The elicitor-induced activity that occurs in roots of sensor plants might well reflect an increase in mature miR168 in these tissues, which would account for the higher level of miR168 accumulation that is observed in elicitor-treated plants compared to non treated plants by stem-loop RT-qPCR analysis (see Figure 1A).In Arabidopsis, the miR168 family comprises two members with identical mature miRNA sequences, miR168a and miR168b. However, neither microarray or stem-loop RT-qPCR analyses, nor the GFP sensor system, provide information on the expression of the individual members of this miRNA family. To investigate the elicitor-responsiveness of each individual family member, we examined the expression of their corresponding precursors (pre-miR168a and pre-miR168b) by qRT-PCR. This study revealed an important increase on the accumulation of both miR168 precursors at 30 min of elicitor treatment which progressively returned to normal values (Figure 3A). These findings support a transcriptional activation of the two miR168 family members, miR168a and miR168b, in response to treatment with fungal elicitors.

It is generally assumed that most *MIR* genes are transcribed by RNA polymerase II and that their upstream regulatory regions contain canonical regulatory elements, i.e. *cis-*elements that are known to regulate transcription of protein-coding genes [7, 49]. Previous studies demonstrated that the *MIR168a* promoter contains numerous abscisic acid-responsive elements (ABREs). Along with this, mature miR168 accumulated in response to abiotic stress and ABA treatment in Arabidopsis [34].

In this work, we scanned the *MIR168* promoter region for the presence of known *cis*-elements related to biotic stress and defence-related hormones. The sequence upstream of the precursor structure for either pre-miR168a or pre-miR168b was extracted from the NCBI database and the transcription start site (TSS) was identified by using the transcription start site identification program for plants (http://linux1.softberry.com/berry.phtml?topic=tssp&group=programs&subgroup=promoter). *cis*-acting elements present in the 2 Kb DNA region upstream of the TSS were searched using the PLACE database (http://www.dna.affrc.go.jp/PLACE/) [50]. Interestingly, the *MIR168a* and *MIR168b* promoters were found to contain several pathogen-responsive *cis*-elements (Figure 3B; Additional file 1: Table S2). Among them, we identified the elicitor responsive element (ERE; TTGACC) as well as several W-boxes, including the WRKY71 and W-boxNPR1 elements. The ERE regulatory element has been shown to direct pathogen- and elicitor-responsive expression in many stress-related genes [51, 52]. W-boxes are the binding sites for SA-induced WRKY transcription factors that are also found in the promoter of the *NPR1* (nonexpressor of *PR* genes1) gene, a key regulator of salicylic acid (SA)-mediated defence responses in Arabidopsis [53]. The SEBF binding site was also identified in the *MIR168* promoter. This regulatory element was initially characterized in the pathogen and elicitor inducible potato pathogenesis-related gene *PR-10a* gene, and later on in the promoter of several other defence-related genes, including *PR* genes. Additional regulatory elements identified in the *MIR168* promoter are associated with defence-related hormones such as ethylene (ET) and methyl jasmonic acid (MeJA) (Figure 3B; Additional file 1: Table S2). To note, several auxin-responsive elements (AuxRE), such as the Auxin Response Factor (ARF) binding site and the Small Auxin-Up RNA (SAUR) binding site are present in the *MIR168* promoter. Furthermore, POBO analysis (http://ekhidna.biocenter.helsinki.fi/poxo/pobo/pobo) allowed us to compare the frequency of each motif identified in the miR168 promoter with the frequency of the same motif in the promoter regions of all known genes from Arabidopsis [54]. Excluding the WboxNPR1, all the pathogen-related *cis*-motifs were significantly enriched in the *MIR168* promoter relative to the Arabidopsis background set (t-test, p-value < 0.0001) (Additional file 2: Figure S1). The SURE, LeCp binding site and GARE elements were also found to be significantly enriched in this promoter. Thus, the observed elicitor-induced accumulation of miR168 is consistent with the presence of pathogen/elicitor responsive elements in the *MIR168* promoter.

**Figure 4:**
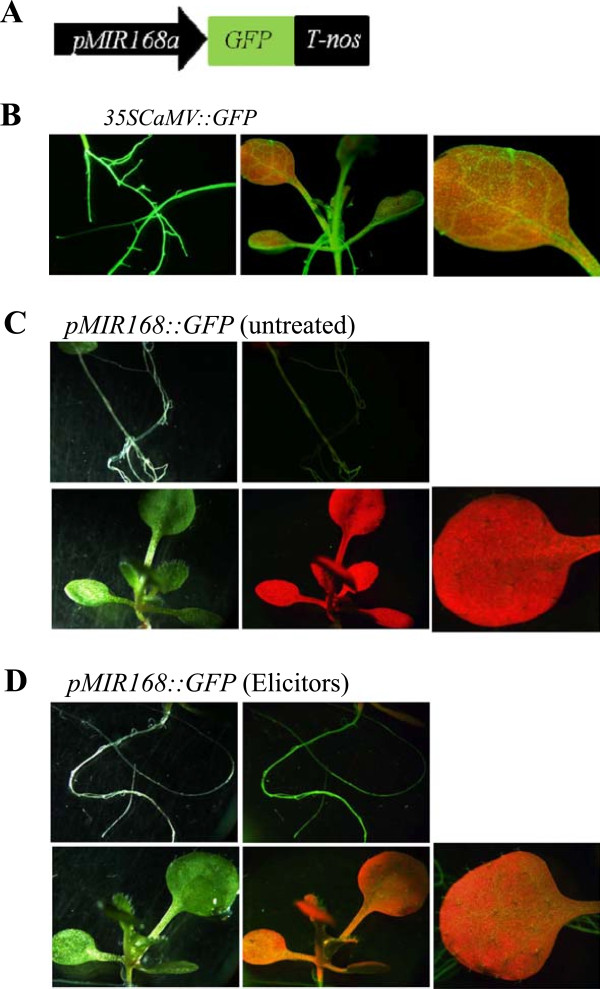
**Functional analysis of the** ***MIR168a*** **promoter in transgenic Arabidopsis. (A)** Schematic diagrame of the *MIR168a* promoter construct. **(B)** Arabidopsis plants constitutively expressing *GFP*. Ten day-old plants were treated with fungal elicitors for 30 min. Water was used as mock control. GFP fluorescence images are shown. **(C)** Control non treated *pMIR168::GFP* plants. Bright-field (left) and GFP fluorescence (middle and right) images are shown. **(D)** Elicitor-treated *pMIR168::GFP* plants Bright-field (left) and GFP fluorescence (middle and right) images are shown.

To further explore the regulatory mechanism of miR168 in the elicitor response, a functional analysis of the *MIR168* promoter was performed, focusing on the *MIR168a* promoter. For this, the *MIR168a* promoter region was fused to the *GFP* gene and the resulting construct (*pMIR168a::GFP*, Figure 4A) was used to transform Arabidopsis plants. As control, transgenic Arabidopsis plants expressing the *GFP* gene under the control of the *35SCaMV* were generated. As expected GFP fluorescence was observed in all tissues of *35SCaMV::GFP* plants (Figure 4B). *GFP* expression could not be detected in tissues of control non treated *pMIR168a::GFP* plants (Figure 4C). When comparing these results with those obtained in GFP-miR168* sensor plants also grown under control conditions (e.g. non treated plants, see Figure 2C) it is concluded that neither *MIR168a* promoter activity nor miR168 activity occurs in non treated roots of GFP-miR168* plants). An apparent discrepancy occurs in leaves of non treated plants as miR168 activity (see Figure 2C), but not miR168a promoter activity (Figure 4C), is observed in these tissues pointing to the existence of additional regulatory mechanisms controlling *MIR168* expression and functioning under normal conditions. In this respect, a transcriptional/translational interlocked feedback loop governing expression of the miR168/AGO1 pair is well documented in Arabidopsis. Indeed, the level of AGO1 mRNA is regulated by both the miRNA miR168 and by siRNAs generated from the AGO1 mRNA after miR168-mediated cleavage [27, 29, 47]. Clearly, further studies are needed to clarify this aspect.

Of interest, GFP was readily detectable in roots of *pMIR168a::GFP* plants that have been treated with fungal elicitors (Figure 4D, left panels) indicating miR168 promoter responsiveness to fungal elicitors, at least in this organ. This finding is consistent with results obtained in GFP-miR168* sensor plants, where the elicitor-induced miR168 accumulation resulted in loss of GFP fluorescence (see Figure 2D). The *MIR168* promoter was also found to be activated in elicitor-treated leaves of *pMIR168::GFP* plants (Figure 4D, right panels). Thus, functional analysis of the *MIR168a* promoter in transgenic Arabidopsis plants confirmed its elicitor-responsiveness while revealing a strong activity of this promoter in roots.

Collectively, results obtained by stem-loop RT-qPCR and miR168 precursor expression analyses, in combination with those obtained in GFP-miR168* sensor plants and *pMIR168::GFP* plants, support that *MIR168* is transcriptionally activated by fungal elicitors to produce active miR168 in Arabidopsis plants.

### siRNA415, an elicitor-inducible hc-siRNA from Arabidopsis

The current release of miRBase contains a sequence annotated as miR415 with two entries, one in *A. thaliana* (aacagagcagaaacagaacau) and another in rice (aacagaacagaagcagagcag). This small RNA sequence was detected by Northern blot analysis in Arabidopsis flowers [47, 55]. However, the status of this sequence as a miRNA has been questioned due to the lack of conservation in genomes other than Arabidopsis and rice, its moderately poor precursor hairpin structure, and the lack of identified targets.

Our microarray analysis revealed a significant increase in the accumulation of this particular small RNA at 30 min of elicitor treatment (FC 5.25; Table 1). Differences in the accumulation of this small RNA sequence between elicitor-treated and control non treated tissues were also distinguished by small RNA Northern blot (Additional file 2: Figure S2). Intriguingly, Northern blot analysis revealed that this small RNA was 24 nucleotides in length, thus, longer than the annotated sequence in miRBase (21-nt in lenght). To further investigate the nature of this small RNA sequence, we monitored its accumulation in Arabidopsis mutants impaired in small RNA biogenesis.

**Figure 5:**
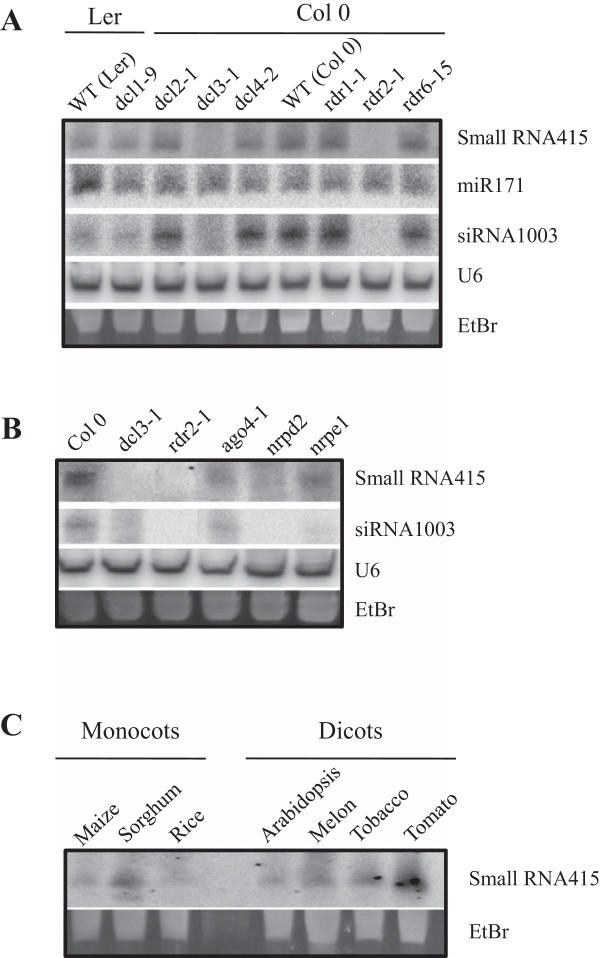
**Genetic requirements for generation of the 24-nt hc-siRNA415. (A)** Analysis of mutants impaired in small RNA biogenesis, *dcl* and *rdr* mutants. The same blot was successively hybridized, stripped, and re-hybridized to oligonucleotide probes corresponding to the complementary sequence of the indicated small RNAs. RNA blots were also probed with the U6 probe for loading control. **(B)** Analysis of *ago4*, *nrpd2* (common to Pol IV and Pol V) and *nrpe1* (Pol V) mutants. **(C)** Small RNA blot analysis of the hc-siRNA415 in different plant species.

Initially, we examined mutants with defects in each *DCL* gene, namely *dcl1-9* (a viable hypomorph), *dcl2-1*, *dcl3-1*, and *dcl4-2*, as well as mutants with defects in a *RDR* gene for which a function has been established, namely *rdr1-1*, *rdr2-1* and *rdr6-15* mutants [7, 14, 56, 57]. Except for *dcl1-9* (Ler background), all the *dcl* and *rdr* mutants were in the Col0 genetic background. The small RNA under study was detected in wild-type plants of the two accessions, its level of accumulation being slightly lower in Ler plants than in Col0 plants (Figure 5A, upper panel). Interestingly, the *dcl3* mutation abolished the accumulation of this small RNA, whereas there were no significant changes on its accumulation in *dcl1*, *dcl2*, and *dcl4* mutants (Col 0 background) (Figure 5A, upper panel). As expected, accumulation of miR171, a canonical miRNA from Arabidopsis, was reduced in the *dcl1* mutant, but not in any of the other *dcl* mutants (Figure 5A).

As previously mentioned, RDR dependencies are characteristic of plant siRNAs. When examining accumulation of the small RNA in *rdr* mutants, its accumulation was found to be compromised in the *rdr2* mutant, but not in the *rdr1* and *rdr6* mutants (Figure 5A).

Evidence exists that 24-nt hc-siRNAs function in RNA-directed DNA methylation (RdDM) in the chromatin silencing pathway. In this work we examined the accumulation of the 24-nt small RNA in Pol IV and Pol V mutants. As previously mentioned, RdDM requires the concerted action of Pol IV and Pol V, the Pol IV being required for hc-siRNA biogenesis (through the RDR2/DCL3 pathway), and Pol V being responsible of targeting siRNAs to loci controlled by RdDM. Pol IV and Pol V each has a unique largest subunit (NRPD1 and NRPE1, respectively) and share the second largest subunit (NRPD2/NRPE2) [22]. As it is shown in Figure 5B, production of the 24-nt small RNA is compromised in the *nrpd2* mutant (second largest subunit, common to Pol IV and Pol V), its accumulation being also significantly reduced in the *nrpe1* mutant (largest subunit of Pol V). Together, results obtained in the analysis of *dcl, rdr,* and *pol* mutants were consistent with the interpretation that the 24-nt small RNA under study is produced by the PolIV/RDR2/DCL3 pathway, a typical feature of hc-siRNAs. Finally, hc-siRNAs are known to function in association with AGO4 [21, 58]. As it is shown in Figure 5B, accumulation of this small RNA was clearly reduced in the *ago4-2* mutant (Col 0 background), this observation supporting that this small RNA associates with AGO4.

As controls, we examined the accumulation of the 24 nt hc-siRNA originating from 5S rDNA (siRNA1003) in the panel of Arabidopsis mutants. Previous studies demonstrated that production of siRNA1003 is Pol IV- and Pol V-dependent [24, 59, 60] and that siRNA1003 accumulation is significantly reduced, or abolished, in *dcl3-1* and *rdr2-1* mutants [7, 18]. Consistent with previously reported results, siRNA1003 was reduced to undetectable levels in *dcl3*, *rdr2*. siRNA1003 production was also impaired in the *nrpd2* (second largest subunit of Pol IV and Pol V) mutant, while showing an important reduction in the *nrpe1* mutant (Pol V largest subunit) compared to wild type Col0 plants (Figure 5B). As expected, siRNA1003 accumulation was not affected in *dcl1-9*, *dcl2-1*, *dcl4-2*, *rdr1-1* and *rdr6-15* mutants (Figure 5A). Thus, siRNA1003 and the siRNA under study exhibit the same genetic requirements of their production, namely Pol IV, RDR2 and DCL3.

Taken together, analysis of Arabidopsis mutants affected in biogenesis and functioning of small RNAs revealed that mutations in any of the genes in the Pol IV/RDR2/DCL3 pathway affect the accumulation of this small RNA species. This piece of evidence strongly supports that the 24-nt small RNA investigated here is indeed a hc-siRNA and not a miRNA. This sequence may therefore be removed in subsequent data releases from miRBase. We have named this small RNA siRNA415.

Finally, we examined whether siRNA415 occurs in plant species other than Arabidopsis. For this, we examined its accumulation in several monocotyledonous (*Zea mays*, *Sorghum bicolour*, *Oryza sativa*), and dicotyledonous (*Cucumis melo*, *Nicotiana tabacum*, *Solanum lycopersicum*) species. As it is shown in Figure 5C, siRNA415 was found to accumulate at different levels in the various plant species examined here.

## Discussion

In this work, we identified a group of small RNAs that are regulated by fungal elicitors pointing to a possible role for these particular sRNAs in PAMP-triggered immunity in Arabidopsis plants. An important number of the elicitor-regulated miRNAs identified in this study are known to control the expression of transcription factors functioning in developmental regulation. This observation further supports a link between plant development and pathogen resistance in Arabidopsis while providing evidence that these miRNAs might be regulated by both developmental cues and biotic stress conditions. Because miRNAs provide quantitative regulation of target gene expression, rather than on-off regulations, the group of elicitor-regulated sRNAs identified in this work might contribute to fine-tune gene expression in reprogramming developmental programs, this process being part of the adaptive strategy of plants to pathogen infection.

Among the set of elicitor-regulated miRNAs here identified, there were several miRNAs controlling genes involved in hormone signaling, namely auxin signaling. In addition to their role in plant development, auxins play an important role in hormone crosstalk during the plant’s stress response [61]. Antagonism between auxin and SA, a major regulator of plant defences, has long been demonstrated [55]. Auxins are also known to regulate the expression of plant defence genes [62]. In other studies, repression of the auxin response pathway was found to increase Arabidopsis susceptibility to necrotrophic fungi [63]. The repression of auxin signaling in Arabidopsis enhances resistance to bacterial infection in Arabidopsis, a process that is mediated by miR393 [40]. Under this scenario, it can be postulated that recognition of fungal elicitors would trigger alterations in the expression of distinct miRNAs which are responsible of reprogramming host developmental processes, including auxin-regulated processes. This perturbation, in turn, might contribute to regulation of defence responses either directly or indirectly through cross-talk between auxin and defence-related hormones.

In our microarray analysis, however, no changes were observed in the expression of the bacteria-responsive miR160 and miR390, also known to control genes involved in auxin signaling pathway. This observation might be indicative of a differential regulation of these miRNAs during infection by either fungal pathogens or bacterial pathogens. Alternatively, the expression of miR160 and miR390 (and perhaps miR393 which was not represented in the microarray) might be regulated at time points of elicitor treatment not assayed in this work. Clearly, regulation of multiple miRNAs exhibiting regulatory links with auxin signaling during PTI responses might provide a fine tune regulation of gene expression in controlling resistance to different types of plant pathogens.

On the other hand, our study revealed that miR168, a miRNA that is deeply conserved among plant species, is transcriptionally regulated by fungal elicitors. Several lines of evidence support this conclusion. Firstly, we demonstrated that mature miR168 rapidly accumulates in response to elicitor treatment. Secondly, expression of miR168 precursors is also up-regulated upon elicitor treatment. Thirdly, functional analysis of the *MIR168a* promoter in transgenic Arabidopsis further supports that *MIR168* is regulated at the transcriptional level. The observed transcriptional activation of *MIR168* is consistent with the presence of various pathogen- and elicitor-responsive *cis-*elements in the *MIR168* promoter. Finally, by using GFP-miR168* sensor plants we demonstrated that the elicitor-induced miR168 is active.

It is well established that miR168 plays a critical role in miRNA functioning by controlling AGO1 homeostasis. Thus, AGO1 homeostasis entails coexpression of *MIR168* and *AGO1* and preferential stabilization of miR168 by AGO1 [3] .In this work, we show that the elicitor-induced accumulation of miR168 leads to a consistent decline on the accumulation of *AGO1* transcripts in elicitor-treated tissues relative to control tissues (i.e. differences between elicitor and control plants progressively diminished during elicitor treatment). On the basis of these data, we can hypothesize that a transcriptional regulation of *MIR168* might be responsible for the control of *AGO1* accumulation early during elicitor treatment. If so, the transcriptional regulation of *MIR168* would represent another layer of control of the refined regulatory system that controls miR168 levels and AGO1 levels. Presumably, an elicitor-regulated adjustment of miR168 levels might contribute to the maintenance of the appropriate levels of AGO1, and accordingly of miRNA functioning, during the plant defense response to pathogen infection.

In the literature there are several reports on the involvement of AGO1 in plant antiviral defense [64]. AGO1 also contribute to flg22-induced disease resistance in Arabidopsis plants [65]. Very recently, Shen et al. [66] described a decrease in miR168 accumulation with a simultaneous increase in AGO1 transcript abundance in roots of oilseed rape (*Brassica napus*) infected with the soil-borne pathogenic fungus *Verticillium longisporum* (at 6 days after inoculation). Furthermore, Weiberg et al. [67] demonstrated that some small RNAs from *Botrytis cinerea* hijack the host RNAi machinery by binding to AGO1 and selectively silencing host immunity responses. Together, these pieces of evidence strongly support that miR168/AGO1 modulation might play an important role in shaping host responses to pathogen infection, including infection by fungal pathogens. In other studies, miR168 was reported to be induced by abiotic stresses (drought, salinity, cold) [34]. Then, it is noteworthy that miR168 is regulated by both biotic and abiotic stress conditions, these findings highlighting the importance of miRNA functioning in plant adaptive processes to environmental stress. Clearly, a fine-tuned adjustment of miR168 and AGO1 levels would provide a flexible system for the control of processes that are critical to ensure plant survival under adverse environmental conditions.

On the other hand, our microarray analysis of Arabidopsis small RNAs allowed us to identify a small RNA (24-nt in length) that transiently accumulates in response to fungal elicitors. Most importantly, analysis of mutants impaired in the small RNA biogenesis pathways revealed that production of this 24-nt siRNA is dependent on the DCL3/RDR2/Pol IV pathway, its accumulation being also reduced by mutations in Pol V and AGO4. As previously mentioned, biogenesis of hc-siRNAs begins with the transcription by RNA Pol IV, which is then followed by RDR2-catalyzed synthesis of dsRNA. Then, si-RNAs (24-nt) are processed from the dsRNAs by DCL3, and loaded into AGO4-containing RISCs. Besides, the function of the AGO4-assembled hc-siRNAs requires the production of a scaffold transcript by Pol V which recruits the AGO4-bound hc-siRNAs. Based on the results obtained in this work, it is concluded that siRNA415 is a *bona fide* hc-siRNA that is dependent on Pol IV/RDR2/DCL3/AGO4 for its biogenesis and function. This small RNA sequence was incorrectly annotated as a miRNA and, accordingly, this entry may be removed from future database releases.

In plants, hc-siRNAs are involved in transcriptional gene silencing by guiding DNA methylation at target genomic loci through RNA-directed DNA methylation (RdDM) [16, 58]. A major function of hc-siRNAs is to maintain genome integrity by silencing transposable elements [68]. Some reports indicate that hc-siRNAs also control the expression of protein-coding genes (i.e. the FLOWERING LOCUS C, FLC gene) [69]. There is then the possibility that siRNA415 might guide DNA methylation at still unknown genomic loci. Further studies are needed to assess whether siRNA415 guides methylation in Arabidopsis plants. Finally, as this small RNA was identified in plant species other than Arabidopsis, both monocotyledonous and dicotyledonous species, there is also the possibility that functioning of this small RNA is not restricted to Arabidopsis.

The scaffolding model for the function of AGO4-associated hc-siRNAs in RdDM implies recruiting DNA methyltransferase enzymes by transcripts being transcribed by Pol V. In this context, DNA methylation in RdDM is maintained by the overlapping functions of Methyltransferase 1 (MET1) and CMT3 in Arabidopsis. Of interest, microarray analysis revealed elicitor-regulation of miR823, this particular miRNA targeting *CMT3* transcripts. The *CMT3* gene encodes CHROMOMETHYLASE3, a protein involved in RdDM. The finding that miR823 is affected by fungal elicitors raises interesting questions concerning the mechanisms by which this particular miRNA might exert its regulatory role in plant immunity by controlling CMT3 expression and RdDM.

Concerning the involvement of RdDM in mediating regulation of plant immune responses, this issue has only recently come to light [70]. Recent reports have shown that DNA methylation is part of the Arabidopsis immune response, i.e. by priming transcriptional activation of some defence genes during antibacterial resistance [70, 71]. A model was proposed to explain regulation of *A. thaliana* immune system by DNA demethylation whereby DNA methylation imparts persistent control over some defence genes during non stressful conditions, but can change dynamically to alter gene expression in response to biotic stress [70]. The contribution of components of the RdDM pathway, such as AGO4 and RNA Pol V, in plant immunity is also documented [72, 73]. Because RdDM can be rapidly reversed by biotic stress, it was proposed that dampening defence gene expression through active RdDM would provide an effective mode of regulation of host defense responses in plants [35].

In summary, results here presented will help in understanding the contribution of small RNAs, both miRNAs and siRNAs, in the Arabidopsis response to pathogen infection while providing new opportunities to elucidate the molecular events controlling plant disease resistance.

## Conclusion

In this study microarray analysis was used to identify small RNAs, miRNAs and siRNAs whose accumulation is regulated by treatment with fungal elicitors in Arabidopsis. Among the elicitor-regulated miRNAs was miR168, this miRNA controlling the level of *AGO1* transcripts. We demonstrated that *MIR168* is transcriptionally regulated by fungal elicitors. These finding suggest that miR168 contributes to the maintenance of the appropriate levels of AGO1, and hence miRNA functioning, in the response of Arabidopsis plants to fungal elicitors. Additionally, we identified an elicitor-regulated hc-siRNA (named siRNA415) which was incorrectly annotated as a miRNA. These results, toghether with the observation that miR823 (targeting CHROMOMETHYLASE3 transcripts) further support a function of RNA-directed DNA methylation processes in mediating plant immune responses. This work represents an effort to identify relevant small RNAs regulating the Arabidopsis response to pathogens that may have relevance to study other pathosystems.

## Methods

### Plant material and treatment with fungal elicitors

Columbia 0 (Col 0) accession of *A. thaliana* was used for this study. Plants were grown on MS0 solid medium at 22 ± 2°C during 15 days under neutral day conditions (12 h light/12 h dark). Fungal elicitors were prepared from the fungus *Fusarium oxysporum* (strain 247) as previously described [42]. For this, the fungus was grown in PDB liquid medium (Potato dextrose, Difco) at 28°C. Fungal mycelium was collected and the whole mycelial suspension was sonicated at 100 W for 15 min, then autoclaved at 115°C (15 psi) for 40 min and finally concentrated by lyophilization. Arabidopsis seedlings were treated with a suspension of fungal elicitors at a final concentration of 300 μg/ml (in sterile water) or mock-inoculated. Plant material was harvested at different time points of elicitor treatment, ground in liquid nitrogen and stored at -80°C. Three independent biological replicates (each one representing approximately 150 plants) were analysed. The following homozygous Arabidopsis mutants were used in this work: *dcl1-9*, *dcl2-1, dcl3-1, dcl4-2*, *rdr1-1, rdr2-1, rdr6-15, ago4-1, nrpd2* (also named *ocp1*), *nrpe1* [21, 24, 57, 73]. The wild-type backgrounds for these mutants were *Landsberg erecta* (Ler, for the dcl1-9 mutant) and Columbia 0 (Col0, for the other mutants).

### DNA constructs and generation of transgenic Arabidopsis plants

The GFP-miR168* sensor construct was prepared as previously described [74]. Essentially, the miR168 target sequence was introduced downstream of the *GFP* coding region by PCR. Amplified fusions were cloned into pGEM-T Easy and then inserted into pBIN61 binary vector under the control of the *35SCaMV* promoter. For preparation of the miR168 promoter-*GFP* construct, a 2 kb fragment upstream of the miR168a precursor was amplified from Arabidopsis genomic DNA and cloned into pMDC110 binary vector upstream of the *GFP* reporter gene. Primers used for PCR are listed in Additional file 1: Table S3. For plant transformation, the DNA constructs were mobilized into *Agrobacterium tumefaciens* strain GV3013 and introduced into Arabidopsis *sde1* mutants (C24 background) [75] by the floral dip method. Green fluorescence was recorded in whole transgenic plants with an Olympus SZX16 stereomicroscope, using 460-nm excitation and 510-nm emission filters coupled to a Digital color camera DP71 Olympus.

### miRNA microarray analysis

For microarray analysis, three sets of RNA samples representing the three biological replicates for each time point (5 min, 30 min, 60 min and 120 min) and condition (control and elicitor-treated) were prepared. Equal amounts of total RNA from each biological replicate were pooled and used for isolation of the small RNA fraction using the miRVana™ miRNA Isolation kit (Ambion®).

The array contained 2,382 probes encompassing: 166 Arabidopsis miRNAs currently annotated in miRBase (http://microrna.sanger.ac.uk), 553 predicted miRNAs, and 1096 small RNA sequences generated by MPSS [45, 46] (Additional file 1: Table S1). In addition, a set of control probes complementary to known *C. elegans* and *D. melanogaster* miRNAs (134 and 78 entries, respectively) were included in the array.

Probe labeling and array hybridization were performed using the NCode™ miRNA Labeling System (Invitrogen, Cat#MIRLS-20), according to the manufacturer´s instructions. Hybridized arrays were scanned with a GenePix 4200A scanner (Axon Instruments, Forster City, California, United States) as previously described [76, 77]. Raw signal intensities for each probe set on each hybridised chip, contained in the .gpr files, were exported from the Gene Pix v.5.1 analysis software (Axon Instruments), and imported into Resolver Gene Expression Data Analysis System v.4.0 (http://www.rosettabio.com) for normalization and error correction. Averages of three biological replicates of normalized values were taken to calculate the expression ratios between elicitors and controls prior to log_10_ transformation. P-values for differential expression calculated by Rosetta Resolver Biosoftware were further adjusted for multi-hypothesis testing using the Benjamini & Hochberg procedure, as implemented in the Bioconductor *multtest* package in R (http://www.bioconductor.org/packages/bioc/stable/src/contrib/html/multtest.html) as described [76, 77]. Probes, for which the adjusted p-value was ≤ 0.05 were considered differentially expressed in the experiment. The microarray data have been deposited in NCBI's Gene Expression Omnibus and are accessible through GEO Series accession number GSE59978 (http://www.ncbi.nlm.nih.gov/geo/query/acc.cgi?acc=GSE59978).

### Gene expression analysis

Reverse transcription reactions were performed using total RNA from Arabidopsis seedlings. The same plant material used for small RNA extraction was also used for total RNA extraction. First-strand cDNA was synthesized from DNase-treated total RNA (3 μg) with SuperScript III reverse transcriptase (Invitrogen GmbH) and oligo-dT_18_ (Qiagen, Hilden, Germany). A specific stem-loop primer was used for miR168 amplification (Additional file 1: Table S3) [78]. The absence of contaminating genomic DNA after DNase1 treatment was confirmed by qRT-PCR analysis using primer pairs designed to amplify a 198 bp intron sequence of the *FLC1* gene. Primer sequences are indicated in Additional file 1: Table S3. qRT-PCRs were performed in optical 96-well plates in a Light Cycler 480 (Roche) using SYBR® Green. Primers were designed using Primer Express software (Applied Biosystems, Foster City, CA, USA). The *Ubiquitin-10* (At5g65080) gene was used as the internal control for normalization. The average cycle threshold (Ct) values from triplicate PCRs were normalized to the average Ct values for the *Ubiquitin-10* gene from the same RNA preparations yielding the ΔCt value. Two independent biological replicates were analysed. T-Student tests were used to evaluate differences in gene expression. Controls of the qRT-PCR reactions without adding the reverse transcriptase enzyme were systematically included in our experiments.

### Small RNA gel blot analysis

Total RNA was extracted using the TRIzol® reagent (Invitrogen™) according to the manufacturer’s instructions. For small RNA Northern blot analysis, total RNAs were fractionated in a 17.5% polyacrylamide gel containing 8 M urea. As probes we used γ^32^P-ATP end-labelled oligonucleotides complementary to the small RNA sequence under study. Probes used for Northern blot analysis are indicated in Additional file 1: Table S3. Blots were pre-hybridized and hybridized in Perfect-Hyb Plus buffer (Sigma). Hybridization signals were detected using a STORM Phosphorimager (GE Helthcare).

### Sequence analysis of *MIR168*promoters

The DNA sequences upstream of the start of the miRNA precursor structure of miR168a and miR168b were extracted from NCBI (http://www.ncbi.nlm.nih.gov). In each case, the 2 kb upstream sequence from the start of the miRNA precursor was scanned for the presence of *cis*-elements using the web-based analysis tool PLACE, Plant Cis-acting Regulatory DNA Elements (http://www.dna.affrc.go.jp/PLACE/). The overrepresentation of known promoter *cis*-elements and motifs was assessed using the POBO application (http://ekhidna.biocenter.helsinki.fi/poxo/pobo/pobo) [54] Statistical significance was calculated using the linked Graphpad application for a two-tailed comparison (P ≤ 0.0001).

### Supporting data

The microarray data obtained in this study have been deposited in NCBI's Gene Expression Omnibus (GEO) database under the accession number GSE59978 (http://www.ncbi.nlm.nih.gov/geo/query/acc.cgi?acc=GSE59978).

## Electronic supplementary material

Additional file 1: Table S1: Arabidopsis small RNAs represented in the microarray. **Table S2.** cis-elements identified in the MIR168a and MIR168b promoters. **Table S3.** Sequences of oligonucleotides used in this study. (PDF 183 KB)

Additional file 2: Figure S1: Frequency of occurrence of defence-related cis-elements in the MIR168a promoter generated in POBO (http://ekhidna.biocenter.helsinki.fi/poxo/pobo/pobo). **Figure S2.** Accumulation of siRNA415 in control (c) and elicitor-treated (e) Arabidopsis plants. (PDF 222 KB)
