# Supplementary material for: Small RNA profiling reveals regulation of Arabidopsis miR168 and heterochromatic siRNA415 in response to fungal elicitors
Source: BMC Genomics. 2014 Dec 10;15(1):1083. doi: 10.1186/1471-2164-15-1083 (PMC4299684; doi:10.1186/1471-2164-15-1083)
Supplement: Supplementary file 1 — Additional file 1: Table S1: Arabidopsis small RNAs represented in the microarray. Table S2. cis-elements identified in the MIR168a and MIR168b promoters. Table S3. Sequences of oligonucleotides used in this study. (PDF 183 KB) [file 12864_2014_6774_MOESM1_ESM.pdf]

Table S1. **Arabidopsis small RNAs represented in the microarray**, including known miRNAs (annotated in miRBase), predicted miRNAs (PRED), and small RNA sequences derived by MPSS [45, 46]. NAO_MPSS, indicates predicted miRNAs identied in MPSS signatures [44]. RAJ-Candidate sequences are from Rajagopalan et al. (2006) [10]. The fold change (elicitor-treated vs non treated plants, p ≤ 0.05) for each miRNA is shown. Three biological replicates and three technical replicates for each biological sample were analysed.

Name	Probe sequence	Direction of miRNA expression			
		5 min	30 min	60 min	120 min
ath-miR156a	gtgctcactctcttctgtca	-	-	-	-1,78932
ath-miR156h	gtgctcactctcttctgtcg	-4,13364	-2,7372	1,40907	-
ath-miR164a	gtgctctctttcttctgtca	-	1,16131	-	-
ath-miR164c	gtgctctctatcttctgtcaa	-	9,50015	-	-
ath-miR165a	tgctttgtctacatttggga	1,31015	-	-	-
ath-miR166a	tgctttgtctacatttgggg	-1,29129	-	-	-
ath-miR167a	tagagctcccttcaatccaaa	-	-	-	-
ath-miR167c	aagagctcccttcaatccaaa	-	-	-	-
ath-miR168a	aggagctcccttcaatccaaa	2,88041	7,56633	2,32473	2,09838
ath-miR169b	ggcatacagggagccaggca	-	-	-	-
ath-miR169d	accccgatgtagtcactttca	-	-	-	-2,32509
ath-miR170	ctggatgcagaggtttatcga	-	-1,75574	-	-
ath-miR172e	tcgaagttccaagtcctcttcaa	-	-	-	-
ath-miR319c	gcacgtgccctgcttctcca	-	-	-	-
ath-miR394a	gcacgtgccctgcttctcca	-	-	-	-
ath-miR404	ggggatgaagcctggtccga	-	-	-	-
ath-miR413	gggaatgaagcctggtccga	-	-	-	-
ath-miR414	tagatcatgctggcagcttca	-	-	-	-
ath-miR415	caagatcatgctggcagctta	-	5,24843	-	-
ath-miR418	cagatcatgctggcagcttca	-	-	-	6,62715
ath-miR774	tcccgacctgcaccaagcga	-	-	-	-
ath-miR775	cggcaagtcatccttggctg	-	-	-	-
ath-miR783	cggcaagtcatccttggctg	1,2665	-	1,85038	1,25286
ath-miR823	ggcaagtcatccttggctca	-	10,65671	-	-
ath-miR832-3p	gccaaggtcaacttgccgga	-	-	-	-
ath-miR833-5p	caggcaagtcatccttggcta	4,5493	3,09978	2,82765	-1,27899
ath-miR834	gatattgacacggctcaatca	-	-	-	-
ath-miR842	gatattggcgcggctcaatca	-	-	-	3,08623
ath-miR844*	cgtgatattggcacggctcaa	-	-	-	-
ath-miR845b	atgcagcatcatcaagattct	-	-	-	-
ath-miR857	gtgaatcttaatggtgctgc	-	-	-	-
ath-miR862-5p	ctgcagcatcatcaagattct	-	-1,72698	-	-
ath-miR863-3p	atgcagcatcatcaagattcc	-	-	-	-
ath-miR864-5p	gtgatttctctctgcaagcgaa	-	-	-	-
ath-miR866-3p	gggagctcccttcagtccaa	-	-	-	-
MAV1	aaggagctcccttcagtccaa	-	-	-	-
MAV3	gcgctatccctcctgagctt	-	-1,67131	-2,13806	-

MAV4	ggcgctatctctcctgcgaa	-	-	-	-2,06731
MAV5	gatcaatgcgatccctttgga	-	-	-	-
MAV7	ggaggtggacagaatgccaa	-1,19144	-1,11853	1,07866	1,52662
MAV8	gagttcccccaaacacttcag	-	-1,06461	-	-
MPSS_b10_102	gagtccccccaaacacttcag	6,84244	15,86732	3,43801	7,55512
MPSS_b10_104	cagttcaagaaagctgtggaa	4,78926	16,03396	-	-
MPSS_b10_106	aagttcaagaaagctgtggaa	-	-50,5391	-	-
MPSS_b10_113	catcaacgctgcactcaatga	-	-	-	-
MPSS_b10_123	catcaacgatgcactcaatga	-	-	-	2,30859
MPSS_b10_127	aaggggtgacctgagaacaca	-	40,11267	-	2,16964
MPSS_b10_141	caggggtgacctgagaacaca	-	-	-4,14465	-
MPSS_b10_142	cagggcaaatctcctttggca	-	-3,95168	-1,42773	-
MPSS_b10_19	agggcaactctcctttggca	-	-	-	-
MPSS_b10_202	ggggcaaatctcctttggca	-	-	-	-
MPSS_b10_206	cgaggcaaatctcctttggca	-	-	-	-
MPSS_b10_21	cgggcaaatctcctttggca	-	5,72729	-4,49082	-
MPSS_b10_222	tttgtgacttataatactctcata	-2,86863	-	-	-
MPSS_b10_230	gtcggtcgacaccagtttcg	-	-	-	-
MPSS_b10_232	cagaggtttaataggcctcgaa	-	-	-	-
MPSS_b10_236	cgagtttgtgcgtgaatctaa	-	-	-	-
MPSS_b10_238	ccgcaaccgccagcgttaat	-	-	-	-1,17021
MPSS_b10_249	agttatgggttagacccaactcat	-	1,00171	-	-
MPSS_b10_25	ttctggattacaatagcattcta	-	-	-3,52979	-
MPSS_b10_252	tttaccaaaagtatatgatttaaa	-	-	-	-
MPSS_b10_255	ccagggaagaggcagtgcat	-	-	-	-
MPSS_b10_256	tgtgcagaacaagagaaactat	-	-	-	-
MPSS_b10_263	ttgacgatgatgatgaagatga	-	-	-	-
MPSS_b10_283	atgttctgtttctgctctgtt	-	-	-	-
MPSS_b10_290	tgaacagtgtacgtacgaacc	-	-4,72117	-	-
MPSS_b10_305	ttcgaacaaattcactaccttc	-	1,61081	-	-
MPSS_b10_31|MPSS_b4_67	tggtcagttcatcatcacatta	3,59561	2,90767	6,89369	1,7651
MPSS_b10_312	tcaacatcctcagcattcataa	-	-	-	-1,15307
MPSS_b10_314	ttgcatttccgtgattagttta	-	-1,08286	-	-
MPSS_b10_329|MPSS_b10_840	tcgtaaggacaaatttccaaaa	-	-	-	-
MPSS_b10_33|MPSS_b4_69	caacaaaacatctcgtccccaa	-	-6,75769	-	-
MPSS_b10_330	caacaaaagatgtcgtccccaa	-	-	1,35593	-
MPSS_b10_346	gtatgggcggagtaggaaaaa	-	-	-	-
MPSS_b10_347	agggctaccacagaggctca	2,22532	-	-59,77222	-
MPSS_b10_357	gagacaaaagctggaagcaaa	-	-	-	-
MPSS_b10_364	gatggccatatgggtaaccaa	-	-	-	-
MPSS_b10_366	ttggcactgctagacatcgaa	1,1339	-	-	-
MPSS_b10_394	tttaacatcaatagaagacttaga	-	-1,63107	-	-
MPSS_b10_395	agcaacgaaactcaatgcgta	-	-	-	-
MPSS_b10_4	cggtgtacataaaccaagcca	-	-	-	-
MPSS_b10_406	atgagcagcaacatagcagaa	-	-	-	-
MPSS_b10_409	acctgctcaacagctgctaga	-	-	1,75893	-

MPSS_b10_419	atgccagatattcacgaagaa	-	-	-	-
MPSS_b10_42	ttttaagtatccagaaaactctaa	-	-	-	-
MPSS_b10_426	aagaacatccaaggtgtttgt	-	-	-	-
MPSS_b10_429	gaacatgaacgagcaaagctt	-	-	-	-
MPSS_b10_438	atgtgcaaatgcttcccgca	-	-	-1,21472	-
MPSS_b10_467|MPSS_b12_97	tatcttatatgatcaccacca	-	-	-	-
MPSS_b10_468	tcccttctcacaaatggtcta	-	-	-	-
MPSS_b10_491	gttcatgcaccttcttgagaa	-	-	-1,19766	-
MPSS_b10_525	cacgtatccaaaaccggacta	-	4,07762	-	-
MPSS_b10_531	tagtttgttgatggtcatctaa	-	-	-	-
MPSS_b10_537	tggaatactcatttaagcaaga	-	-	-	-
MPSS_b10_540	attccatcatttggtatcagagct	-	-	-	-
MPSS_b10_557	ttctaccttgaagctttaatttg	-	-	-	-
MPSS_b10_580	tttcacttcttctcaaaatagtta	-	-	-	-
MPSS_b10_587|MPSS_b10_908	caagaagagtacgaagagatca	-	-	-	-
MPSS_b10_593	cttgcttggattgggaatcaa	-	-	-	-
MPSS_b10_600	tttcgattcccgatcccagca	-	-	-	-
MPSS_b10_603	cttgtttgttgacatcggtcta	-	-	-	-
MPSS_b10_609	actagaccgagtacaacaaaca	-	-	-	-
MPSS_b10_613	ttaccaccgctactgctacca	-	-	-	-
MPSS_b10_62	ctttcttgcgtatcttctcca	-	2,32526	-	-
MPSS_b10_633	tttgataaagaacatatgcaagaa	-	-	-	-
MPSS_b10_641	cgcatcaaaggaaacacagga	-	-2,76372	-	-
MPSS_b10_644	tccatcagttttttgttcgttt	3,06847	3,35873	-	-
MPSS_b10_652	ttgaaacgaacaagaaactgat	-	-	-	-
MPSS_b10_66	ttgtgcaagaagtagaagaaaa	-	-	-	-
MPSS_b10_671	gggaacgatgaaaggttggta	-	-	-	-
MPSS_b10_691	gttagtttaggtccttcagtgt	-	-	-	-
MPSS_b10_693|MPSS_b4_56	ttcagtttcaagtggctcgta	-	-	-	-
MPSS_b10_696	ggatgacggatctgaccatga	-	-1,80126	-	-
MPSS_b10_70	tccaatgaagctcgacctaaa	-	-	-	-
MPSS_b10_715	tagcttataagcaatcttacca	-	-	5,01129	-
MPSS_b10_716	tttaactagtaagatggcttataa	-	-1,3786	-	-
MPSS_b10_756	catcaattggtatcagagccg	-	-	-	-
MPSS_b10_758	catcaatttggtatcagagcga	-	1,61559	-	-
MPSS_b10_759	taattcaagcacttcaattcaa	-	-1,48968	-	-
MPSS_b10_76	catcaagaagaagaggagtga	1,86998	-	-	-
MPSS_b10_762	agcttaggcagtcccatgtca	-	1,44013	-	-
MPSS_b10_771	ttttactacaccaatgtttagtta	-	-	-	-
MPSS_b10_774	ctttgttgtagtccggatctta	-	-	-	-
MPSS_b10_776	gtcgtctttgtttgccaccca	-	1,18308	-	-
MPSS_b10_777	ttgtggatcgcgaaccgaga	-	-1,48419	-	-
MPSS_b10_78	cagaactaaggcgcttatctt	-	-1,65647	-	-
MPSS_b10_787	cttctccaagctaaagaggga	-	-1,38229	-	-
MPSS_b10_789	ctcctcctccctatcctcatc	-	-2,08102	-	-
MPSS_b10_790	ttccttttccttagcttttgct	-	-1,39953	-	-

MPSS_b10_802	gctgaagttattggtaggatta	-	1,80368	-	-
MPSS_b10_803	tttatacaccttcaacatacaaaa	-	-	-	36,16347
MPSS_b10_806	aaggtcgaacagacaacgaaa	-	-	-	-
MPSS_b10_820	tttgacttcacaacagagaga	-	-1,54206	-	-
MPSS_b10_821	tttatacatagtccaatctattga	-	-2,47485	17,05781	-
MPSS_b10_825	tgtccttgaagacatatccatc	-	-2,52308	-	-
MPSS_b10_831	ttgacgcatatttctccaagg	-	-	-	-
MPSS_b10_835	tcttcaagtagatccagcatat	6,98886	2,27121	1,96308	-
MPSS_b10_836	gcacatgctcgacctattgga	-	-	2,07133	-
MPSS_b10_843	tattatgtcttgttgctctcaa	-	-	-	-
MPSS_b10_856	tttattgagatcaacaagacataa	24,38552	73,4314	-	-
MPSS_b10_861	ttcttcaaggtattattgacttta	-	-	-	-
MPSS_b10_862	ttttgaagtcaatcatacctga	-	1,7324	-	-
MPSS_b10_863	tttttggataaatttgaggaaaaa	-	-	-	-
MPSS_b10_877	tttctcaattagatccaaattcat	-	-	-	-
MPSS_b10_879	tttcttcaaagacggattttgt	-	-3,19289	-	-
MPSS_b10_889	tttaacaaaatccgttccttga	-	-	-	-
MPSS_b10_896	ttttcctaataaaccatgttcaa	-	-	-	-
MPSS_b10_90	tgcattatcagcacttaagaag	-	-	-	-
MPSS_b10_903	caacaccagaattgaaccaat	-	-	-	-
MPSS_b10_919	gtcaactatctcaacaccaga	-	-	-	-
MPSS_b10_92	tgatcgaagaaacaccaaatta	-	1,39725	-	-
MPSS_b10_923	tatccgcttcgtgcccagcc	-	-	-	-1,07542
MPSS_b10_928	ttttgtcaaattagacaaaatttc	-	-	-	-
MPSS_b10_93	gtttgggcacgaagcggata	-	-	-	-
MPSS_b10_950	atggtgatcgaaccgcacaat	-	-	-	-
MPSS_b10_952	tcgaaccgcacaatgaaatac	-	-	-	-
MPSS_b10_957	cttcgcaggagagatagcgc	-	-	-	-
MPSS_b10_96	tgtgaaaactaggtttgggca	-	1,37381	-	-
MPSS_b10_97	agaaaccctcaaccgccaga	-	-	-	-
MPSS_b11_33	ttcgtttggattgaagggagc	-	-1,04427	-	-
MPSS_b12_1	tatcctcggaccaggcttcat	-	-1,93124	-	-
MPSS_b12_17	agctgaagctgccagcatga	-	-	-	-
MPSS_b12_45	gtttggactgaagggagctcc	-	-2,62854	-	-
MPSS_b12_67	tttttactacaaactttttatgct	-	-	2,74972	-
MPSS_b12_73	tatgtttctggcaagaaagaag	-	-	-	-
MPSS_b12_84	cgtcggaccaggcttcattc	-	-	-	-
MPSS_b15_1769	gtgaagaagaatggattcccc	-	3,42422	-	-
MPSS_b15_1817	caaacgatcatgttctcctga	-	-	-	-
MPSS_b17_10	gattgagccaaggatgacttg	-	-1,87361	-	-
MPSS_b17_16	atactacgcctccaattgaac	-	-	-	-
MPSS_b17_17	tactacgcctccaattgaacc	-	-	-	-
MPSS_b17_20	tgcagccaaggatgacttgc	-	-	-	-
MPSS_b17_32	ttcccgtaatatagcaaagatc	-	-	-	1,20985
MPSS_b17_33	tgtcccgtaatatagcaaagat	-	-	-	-
MPSS_b17_42	atccttttcgtgatctttgct	1,12326	1,41782	-1,33031	1,28149

MPSS_b17_47	gacaaaagggtggtccgtgat	-	-	-	-
MPSS_b17_52	tgaagagagttgaccaacttt	-	-	-	-
MPSS_b17_61	agagagcttccttgagtccat	-	-	-	-
MPSS_b17_63	gattggactgaagggagctcc	-	-	-	-
MPSS_b17_73	tgattctcggaccaggcttca	-	6,83389	-	-
MPSS_b17_75	attctcggaccaggcttcatt	-	-	-	-
MPSS_b17_76	ttctcggaccaggcttcattc	-	-	-	-
MPSS_b17_9	attgagccaaggatgacttgc	-	-	-	-
MPSS_b3_17	agggctaccacagaggctca	-	-	-	6,18778
MPSS_b3_22	tatcgaagtgtacgaatcaaac	-	-	-	4,13532
MPSS_b3_3|MPSS_b9_1	tatccaagtgtacgaatcaaac	-	-	-	-
MPSS_b3_34	gctacgtaggagagataccgt	-	-	-3,10097	-
MPSS_b3_4|MPSS_b9_38	tttatctaagtgtacgaatcaaac	-	-	1,6327	-
MPSS_b3_7	gggaacgatgaaaggttggta	-	-	-	-
MPSS_b4_17	aaaacagcgttattgcaaaga	-	-	-	-
MPSS_b4_44	ccgggaaaaaaaattgtgtct	-	-	22,76	-
MPSS_b4_76	ccgcaaccgcaaacgcttgc	-	-	-1,28996	-
MPSS_b4_77	cccgcaaccgcaaacgcttg	-	-	1,67221	-
MPSS_b7_25678	gttcgaggattcgtcgaccag	-	-	-10,12671	1,90304
MPSS_b7_37064	cgctacgccatcgcccgaga	-	-	-	-
MPSS_b7_37969	ccgcaactacctgcggcgac	-	-	-	-
MPSS_b7_4076	aaatccatccgccaccgagg	-2,10808	1,21144	-1,5758	-1,14388
MPSS_b8_11	ctcttctttggattgaaggga	-	-	-	-
MPSS_b8_111	agggcgcttaggctggctga	-	-2,68066	-2,28945	-
MPSS_b8_127	ggaactagataaggcccgcct	-	1,45107	-	-
MPSS_b8_13	tctttggattgaagggagctc	-	-1,37085	-	-
MPSS_b8_131	atacctatgtggacaacgact	-	-	-	-
MPSS_b8_137	atggccaagtcaacgaaactt	-	-	-	-
MPSS_b8_140	atgtccagatgggttcgcct	-	-	-	-
MPSS_b8_155|MPSS_b9_37	ttaaacacaagccgcacttga	-	-	-	-
MPSS_b8_166|MPSS_b9_36	gcatcatgccagatagagccc	-	-	-	-
MPSS_b8_168	tcatatattgaacctggcacac	-	-	-	-
MPSS_b8_177	agctgcttgatccgcctgat	-	-	-	-
MPSS_b8_181	gtaacagccacgcttggtga	-	-	-	-
MPSS_b8_185	aaagtgtgtcacgatacgctt	-	-	-	-
MPSS_b8_195	ccaaaggtgttccggcggtt	-	-	-	-7,4945
MPSS_b8_216	atatgtgcattagtgctggta	-	-	-	-
MPSS_b8_217	tatgtgcattagtgctggtat	-	-	-	1,28096
MPSS_b8_22	acatgtggcataagtctcatt	-	-	-	-
MPSS_b8_226	gatccacataaggcccgcct	-	-	-	-
MPSS_b8_245	cttggtatcagagccaggttt	-	21,05574	-	-
MPSS_b8_247	cgcaccaaataccgttggct	-	-	-	-
MPSS_b8_26	atattggtcccaactcgggac	-	-	-	-
MPSS_b8_261	aaaggtggtccaagggaaaca	-	-	-	-
MPSS_b8_279	atcggtcccaagtcgggcca	-	1,11196	-	-
MPSS_b8_291	atgcagtgtcttggcaactct	-	-	-	-

MPSS_b8_298	cgtggcacaatcctatgagtt	-1,39269	-	-	-
MPSS_b8_302	tttgacacgtggcacaatcct	-	-	-	-
MPSS_b8_312	tctaaccgatggagatggtct	-	-	-	-
MPSS_b8_315	agagattacggacttcgcctt	-	-	-	-
MPSS_b8_325	tactaattggcgaaaaatgtgt	-	-1,40936	-	-
MPSS_b8_335	tggcagccattgtaaataagt	-	-	-2,01339	-
MPSS_b8_339	ggagaagccacgtcagatgat	-	-	-	-
MPSS_b8_49	ggccagttccgtgtcctaat	-2,34042	-1,81113	-	-
MPSS_b8_58	catcagcacatgagagtttgc	-	-	-	-
MPSS_b8_8	tatactagataaggcccgcct	-	1,17345	-	-
MPSS_b8_81	gttgaagaggacttggaactt	-	-	-	-
MPSS_b8_87	caaggtcgaacagacaacgaa	-	-1,61809	-	-
MPSS_b8_89	aaggtcgaacagacaacgaaa	-	-2,03007	-	-
MPSS_b8_98	aggtggtggcggctgtcgta	-2,14592	-1,21086	1,34466	1,34111
MPSS_b9_29	gttaagctgccagcatgatct	-	-	-	-
NAO_MPSS_b15_4386	gcacttctcccgcaccgcaa	-	-	-	-
NAO_MPSS_b7_13322	caacaatgtagagctcgtcga	-	-	-	-
NAO_MPSS_b7_15340	gacgaagccgatgtcgttga	-	-	-	-
NAO_MPSS_b7_16927	aggtcttgcaaggtcaagaaa	-	-	-	94,78667
NAO_MPSS_b7_16928	gggtcttgcaaggtcaagaaa	-	-	-	-
NAO_MPSS_b7_19312	aagcattgatcatcatcgcca	-	-	-	-
NAO_MPSS_b7_20142	agatccagacgcggcttcca	-	-1,48925	-	-
NAO_MPSS_b7_22139	gagctccaccaagtcatcaga	-	-	-	-
NAO_MPSS_b7_23469	gtgttcctgtttcatctctca	-	-13,32689	-	-<br>25,78215
NAO_MPSS_b7_25417	ggctccaacagtctcagctaa	-	-	-	-
NAO_MPSS_b7_26344	tatttcctgctcccaagttaa	-	1,86868	-	-
NAO_MPSS_b7_27131	tgggaaattttcagtttccaat	-	-	-	-
NAO_MPSS_b7_27720	gagctgttggtcctccacca	1,42556	-1,24279	-1,55315	-1,28106
NAO_MPSS_b7_32204	ggcctgacccatctttgcag	2,36694	4,39246	6,67721	-1,56724
NAO_MPSS_b7_33589	taggtagatgtgctgattcga	-	2,42005	-	-
NAO_MPSS_b7_34280	ccaccgacatgctcacgaaa	-	1,04901	-	-
NAO_MPSS_b7_3521	gaggcaaagcaaggtcacgaa	-	1,19682	-	-
NAO_MPSS_b7_355	cgccaactttcagtttccaat	-	-	-	-
NAO_MPSS_b7_37837	gcgccattcaacctccctca	-	-	-	-
NAO_MPSS_b7_40315	tggtttaaccatctccatcaa	-	-	-	-
NAO_MPSS_b7_40631	ggcctgacccatctttgcag	4,66134	5,41404	5,84646	-1,19621
NAO_MPSS_b7_40639	tggtcttttgttcttctctctg	-	1,93579	-	-
NAO_MPSS_b7_8451	tgaacctttgttcttgagctaa	-	-	-	-
NAO_MPSS_b7_8746	gatcaagcatagcggatcaaa	-	-	-	-
NAO_MPSS_b7_8943	aggcacgagaccaatcttcga	-	-	-	-
PRED_103783|456	tcacatgggaagggagaggc	-	-	-	-
PRED_104245|431	tcgtcgtctccttcaagatctc	-	-	-	-
PRED_113736|251	acccacaaattccgggtcca	-	-1,23569	-	-
PRED_114143|241	cacctgagcgagccaacttg	1,76742	1,17898	1,6714	-1,01493
PRED_114300|268	agtcgtccagccgcaagagt	-	-11,59434	-	-
PRED_116412|60	tccagacctggaatcgatcg	-	-	-	-

PRED_116413|73	gaatcgatcgatcccgagct	-	-1,48042	-1,81494	-
PRED_124666|252	tcgaccaacttgtactatgcc	-	-	-	-
PRED_126615|281	attgcagctctatcagttgtt	-	-	-	-
PRED_132308|3	agcggctgcggcaacgttgg	-	1,99461	-	-
PRED_132311|25	gctgccgcaaccgcagccgc	-	-	-	-
PRED_132313|25	cgcagccgctgccgcctgcg	-	-	-	-
PRED_138713|201	acagagagatatggaaggaagtt	-	-	-	-
PRED_14323|452	ccaagcactgaaacattccac	-	-	-25,43076	-
PRED_143934|83	aagggtgaggtcttgggttc	-	-	-	-
PRED_143936|142	cttggcgttcctcgaaccca	-	-	-2,20845	-
PRED_145225|204	ttgaaaatggaaacctgaaagg	-	-	-	-
PRED_149717|165	ttcaactagaggagcgacca	-	-92,61353	-	-
PRED_149718|161	ggggtattccgaaggtgtaac	-	-	-2,68724	-
PRED_154298|156	cgctcctggattgtcggtct	-	-1,43231	-	-
PRED_169767|160	agccatcaccaaagccacct	-5,56603	-1,66017	-1,68151	-
PRED_169771|203	cggttgcggctgagtctatc	7,09388	-	2,57096	1,07513
PRED_170335|238	ggcttacggttgtggttctca	-	-1,96509	-	-
PRED_178184|222	aggaggacgcctcttcttct	-	-	-	-
PRED_178728|45	agaggaagcaagaggatgtg	-	-	-	-
PRED_193546|190	gcctgacaacaatattcgttag	-	-	-	-
PRED_197522|209	gttgccactgactccaccag	-	3,45727	-4,02784	-
PRED_199777|249	tctctttctcccaccgtctc	-	-2,07573	-	-
PRED_201718|176	caccactagagcactctccag	-	-	-	-
PRED_201721|88	cagccgggagagaagagctc	-	-	-	-
PRED_201991|244	ggaaccgagccaactaaacaa	2,60525	1,51123	-1,16222	-1,3292
PRED_204872|56	ccgaggattcgtcgaccagg	-1,60833	-2,34815	-2,27826	1,66669
PRED_204927_205064a	gaatcgtcgaccaggacgag	3,58354	1,00382	1,86557	1,58775
PRED_204927_205064b	gaatcgtcgaccaggacgag	-	-	1,88961	-
PRED_204949|56	ccgaggattcgtcgaccagg	-1,51109	-1,83389	-3,65677	1,98263
PRED_206190|280	cactcgaccgacgccgcctc	3,25964	1,70606	-	1,11516
PRED_20629|422	gcagaccagtgcgctacaag	-	-	-	-
PRED_209045|109	acctaacgagcagtaccttgtt	-	-	-	-
PRED_20922|44	ttccaatgttaggtctagattg	-	2,14782	-	-
PRED_211873|218	ttagctcagaagaatcaagtga	-	2,52175	-	-
PRED_212929|278	tcatcaccttggaatctctct	-	2,0604	-	-
PRED_215958|82	taccccttcctccgattttc	-1,1738	-1,68562	-1,1628	-1,10834
PRED_222700|211	acatcctcttgcttcctctcc	-4,1113	-	-	-
PRED_222812|199	tttgaagcaaaattgtcatgga	-	-	1,31408	-
PRED_232477|54	gcgaagaaatcgtcggatgtc	-	-	1,14109	-
PRED_241107|48	tcgagctagtggagctggcc	-	-1,4209	-	-
PRED_24693|122	gccaccaccaccgccggtga	1,0975	-1,10991	-1,02194	1,09373
PRED_24694|36	ccaccaccgccggtgaccat	-1,51617	1,01563	-1,22515	-1,22665
PRED_248355_248385_248468a	cctcgaccactcctcggaca	-	-	-1,15249	-
PRED_248355_248385_248468b	cctcgaccactcctcggaca	-	-	5,97182	-
PRED_248388|111	tggtcgacgaatcctcggac	1,76397	-	-	-
PRED_252331|109	gtgtttgaacctaacgagcagtac	-	-1,03436	-	-

PRED_254322|264	tctacggcactcacgccgtt	-	-	-	-
PRED_254844|50	cacaggaacatgatgatggc	-	6,21501	-	-
PRED_257133_257146b	tattggcgatggccctttgc	-	-	-	-
PRED_257983|129	gtgtgggtggcaaacaaaga	-	-	-	-
PRED_259051|31	ctccgccgtgttcttccact	-1,41214	-1,09183	-1,32736	-1,09584
PRED_25939|121	atccgccaccgagggattga	-2,22465	-1,59266	-2,15387	-2,4462
PRED_25969|121	atccgccaccgagggattga	1,19212	-1,54086	-1,46316	1,06681
PRED_26386|442	caaggtcgcccatgcagcac	-	-	-	-
PRED_264979|39	atcctcgaccaggacaaagaat	-	-2,28751	-	2,09442
PRED_276194|324	aggatagaggagaaaccagta	-	-	-	-
PRED_277863|315	aaattcttcgggttcatgcgg	-	-3,50047	-47,10359	-28,76945
PRED_282348|368	tgaattcccacagacggcgc	-	-4,02797	-	-
PRED_290915|292	caaacacagagaccctcctcc	-2,60619	-1,00277	-1,44813	-1,32343
PRED_298202|311	ttccaccaccgcaacagcca	-1,24749	1,04257	-1,47885	1,15796
PRED_304982|319	gatcgatctgctcctcagat	-	-	-	-
PRED_306241|340	cactcgaccgggaagagcaa	-	-	-	-
PRED_310417|34	aagaaggtgagacttgcatgaat	-	-	-	-
PRED_324326|342	ccttctctcccgccgacgat	-	-	-	-
PRED_333080|9	ggtgaggataaccatcggggt	-	-	-	-
PRED_337312|140	cactcctacaccgtcatcca	-	1,11667	-	-
PRED_344773|346	tcctgtctccaccagctccc	-1,54925	1,15312	-1,43749	-1,18027
PRED_350066|42	tgccacgtggcatcctgttg	-	2,07791	-	-
PRED_351492|297	ctctctccaacccgaagccc	-	-	-	-
PRED_352028|322	cgtccgtaccacgggtcatc	-	-2,23026	-	-
PRED_352263|345	ccgaactggggaacatttttttt	-	-	-	-
PRED_352831|351	caggcataccgcggatgtac	-	-	-	-
PRED_361446|294	tccatcgccgacgccaccac	-1,05483	1,29518	-1,70235	1,02433
PRED_368659|8	accaacactttctcgtccctt	-	-2,11092	-	-
PRED_369847|0	ctccatctccgtctcttttcc	1,13865	-1,0166	-1,6982	-1,76368
PRED_370006|286	tcttcctccgatccgatctg	-	-	-9,71546	-
PRED_379984|336	ttagaagcaaagagaaagttgc	-	-	-	-
PRED_38895|408	gccgattagcctctcttcctc	-	-	-1,42327	-
PRED_39260|113	aagcgggatgatgaatccgac	-	-	-	-1,90178
PRED_39263|91	caccatcatggtcagatccg	-	1,76506	-	-
PRED_395402|387	tccctagagaaggcaaacaa	-	-	-	-
PRED_406024|96	ctcaccgtttctcctccgcc	-1,18464	-1,46439	-2,30873	1,2668
PRED_410213|314	gctgctggacgtgaagcctc	-	-	-	-
PRED_423107|287	ctcaacaacctcgcttcacc	2,02204	2,77329	2,37594	-1,01694
PRED_42713|418	agtcacctccggggttcttc	-	-	-	-
PRED_45664|454	aaccaaacaataccgggccac	-	2,33706	-	-
PRED_55822|90	atcctccaccgtcatcctcg	-2,01383	1,21368	-1,2152	-1,17209
PRED_55823|90	aatcctccaccgtcatcctc	-2,94746	-1,04926	-1,40299	-1,01457
PRED_63677|436	tctcttctgctttctgtcaaacg	-	-	-	-
PRED_72283|86	cttctagtcaccaccacgtc	-	2,91582	-	-
PRED_75065|409	gtccagttcgaggattcgtcg	-	-	-4,19193	-
PRED_87782|80	accgtcgccttcaccgcaag	-4,8981	-3,12579	-1,05159	-

PRED_87783|80	ccaccgtcgccttcaccgca	-1,18079	-1,24681	-1,59326	-1,05991
PRED_92544|46	cggatctgaccatgatggtg	-	-	-	-2,22093
PRED_At1g25460_26	tggtgattgtgacttgtgaa	-	-	-	-
PRED_At1g32200_463_rc	aaatcatgtgacgtcgacaac	-	-9,14249	-	-
PRED_At1g40136_5_24	aaccgtaaaccctaaaccata	-	-2,62781	-1,16992	1,72537
PRED_At1g61840_4217	tagaagaaaaagagagtcatgg	-	-1,38191	-	-
PRED_At2g05580_5_4	tcaatcgaaattctctgactac	-	-	-	-
PRED_At2g28470_1616_rc	ttccaaacctacaaagtacaaa	-	-	-	-
PRED_At4g38520_371_1	gctccacttccctctcgtcc	-1,393	1,0904	-1,38473	-1,41452
PRED_At5g25180_5_12	atttttgggtatggatggaca	-	-	-	-
PRED_At5g38490_130_1	gactccactgttttctgcatt	-	-	-	-
PRED_mcat2	ttttcaaaaacactatctcttttg	-	-	-	-
PRED_mcat25b_mcat25b-star	acttatattatgaaacagagggag	-	-	-	-
PRED_mcat31_mcat31ov	aatactccctcagtttcacaat	-	-	-	-
PRED_mcat37	tccaagttggtattaccaacc	-	-	-	-
PRED_mcat55b	tttcccttcgtttcataatataag	-	-	-2,85399	-
PRED_mcat79	actccctccgtttcattataagt	-	-	-	-
PRED_MIR-12	ttttcttaaaatgcttctatgaga	-	-	-	-
PRED_MIR-19	gtacaggttcttctcctcctca	-1,07918	-1,24175	-1,50251	-1,30571
PRED_MIR-36	ttaactgtaacgtaccgataaa	-	-	-	-
PRED_MIR-40	atcatcatcatcgtcattgca	-5,2349	-1,4514	-1,07138	-2,519
PRED_MIR-41	ttttattagtaaaccatggtaaga	-	-	5,56426	-
PRED_MIR-46	tatggaaatctctctctcttc	-	-1,70993	1,01356	-
PRED_MIR-51	ttcaagactcctcttcttcttc	-	-1,6257	-	-
PRED_MIR-52	ttttgtaaactcgaaaacactc	-	-	-	-
PRED_MIR-56	tttgttgtacatgtacgaaaaa	-	-	-	-
PRED_MIR-57	tcaaaaaacgaatggttccaaa	-	-	-	-
PRED_MIR-59	taaagttgatgcatttgtacct	-	-	-	-
PRED_MIR-81	ctcgtcgtcatcatcgtcttc	-10,67857	1,32202	-5,61251	-
PRED_MIR-82	gtagtacattcaaaagtcaacaa	-	-	-	-3,87769
PRED_MIR-9	tttttctctcgaagaagaagaa	-	1,57451	-	-
RAJ-CandidateAF	gtcaaggtcaggggcattaca	-	1,28622	-	-
RAJ-CandidateAK	gacaccaaagatgagttcga	-	-	-	-
RAJ-CandidateAM	tccacctgaaggtcaagctca	-	-	-	-
RAJ-CandidateAN	gtcaaggtcaggggcattaca	-	-3,53402	-	-
RAJ-CandidateL	tttttctttgcttgaaattgttaa	-	-	-	-
RAJ-CandidateM	aataaatcccaaagcatcttcca	-	-	-	-
RAJ-CandidateS	cgcttcccttgaccaatcca	-	-	-	-
RAJ-CandidateV	gtcttgttgctctcaatcgca	-	-	-	-
RAJ-MIR772-5P	tcctacttcgaccatacataca	-	-	-	-
Controls					
Mut-miR156a	gtgctcacAcActtctgtca	-	-	-	-
Mut-miR156g	gtgctcacAcActtctgtcg	-	-	-	-
Mut-miR156h	gtgctctcAtActtctgtca	-	-	-	-
Mut-miR157a	gtgctctctaAcAtctgtcaa	-	-	-	-
Mut-miR158a	tgctttgtcAaGatttggga	-	-	-	-

Mut-miR158b	tgctttgtcAaGatttgggg	-	-	-	-
Mut-miR159a	tagagctccGtAcaatccaaa	-	-	-	-
Mut-miR159b	aagagctccGtAcaatccaaa	-	-	-	-
Mut-miR159c	aggagctccGtAcaatccaaa	-	-	-	-
Mut-miR160a	ggcataGagggagGcaggca	-	-	-	-
Mut-miR161	accccgatgAagAcactttca	-	-	-	-
Mut-miR162a	ctggatgGagaggAttatcga	-	-	-	-
Mut-miR163	tcgaagttcGaagAcctcttcaa	-	-	-	-
Mut-miR164a	gcacgtgcGcAgcttctcca	-	-	-	-
Mut-miR164c	gcacgtgcGcAgcttctcca	-	-	-	-
Mut-miR165a	ggggaAgaagGctggtccga	-	-	-	-
Mut-miR166a	gggaaAgaagGctggtccga	-	-	-	-
Mut-miR167a	tagatcatgGtggGagcttca	-	-	-	-
Mut-miR167c	caagatcaAgGtggcagctta	-	-	-	-
Mut-miR167d	cagatcatgGtggGagcttca	-	-	-	-
Mut-miR168a	tcccgaccAgGaccaagcga	-	-	-	-
Mut-miR169a	cggcaagtGaAccttggctg	-	-	-	-
Mut-miR169b	cggcaagtGaAccttggctg	-	-	-	-
Mut-miR169d	ggcaagtcaAcGttggctca	-	-	-	-
Mut-miR169g*	gccaaggtGaaGttgccgga	-	-	-	-
Mut-miR169h	caggcaagtGaAccttggcta	-	-	-	-
Mut-miR170	gatattgaGaGggctcaatca	-	-	-	-
Mut-miR171a	gatattggGgGggctcaatca	-	-	-	8,62278
Mut-miR171b	cgtgatatAggGacggctcaa	-	-	-	-
Mut-miR172a	atgcagcatGaAcaagattct	-	-	-	-
Mut-miR172b*	gtgaatctAaaAggtgctgc	-	-	-	-
Mut-miR172c	ctgcagcatGaAcaagattct	-	-	-	-
Mut-miR172e	atgcagcatGaAcaagattcc	-	-	-	-
Mut-miR173	gtgatttctGtGtgcaagcgaa	-	-	-	-
Mut-miR319a	gggagctcGcAtcagtccaa	-	-	-	-
Mut-miR319c	aaggagctcGcAtcagtccaa	-	-	-	-
Mut-miR390a	gcgctatcGcAcctgagctt	-	-	-	-
Mut-miR391	ggcgctatGtGtcctgcgaa	-	-	-	-
Mut-miR393a	gatcaatgGgaAccctttgga	-	-	-	-
Mut-miR394a	ggaggAggacagaaAgccaa	-	-	-	-
Mut-miR395a	gagttccccGaaaGacttcag	-	-	-	-
Mut-miR395b	gagtcccccGaaaGacttcag	-	-	-	-
Mut-miR396a	cagttGaagaaagGtgtggaa	-	-	-	-
Mut-miR396b	aagttGaagaaagGtgtggaa	-	-	-	-
Mut-miR397a	catcaacgcAgGactcaatga	-	-	-	-
Mut-miR397b	catcaacgaAgGactcaatga	-	-	-	-
Mut-miR398a	aaggggtgaGcAgagaacaca	-	-	-	-
Mut-miR398b	caggggtgaGcAgagaacaca	-	-	-	-2,23033
Mut-miR399a	cagggcaaaAcAcctttggca	-	-	-	-
Mut-miR399b	agggcaacAcAcctttggca	-	-	-	-
Mut-miR399d	ggggcaaaAcAcctttggca	-	-	-	-

Mut-miR399e	cgaggcaaaAcAcctttggca	-	-	-	-
Mut-miR399f	cgggcaaaAcAcctttggca	-	-	-	-
Mut-miR400	tttgtgactAaAaatactctcata	-	-	-	-
Mut-miR401	gtcggtcgaGaGcagtttcg	-	-	-	-
Mut-miR402	cagaggttAaaAaggcctcgaa	-	-	-	-
Mut-miR403	cgagtttgtgGgAgaatctaa	-	-	-	-
Mut-miR404	ccgcaacGgGcagcgttaat	-	-	-	-
Mut-miR405a	agttatgggtAagaGccaactcat	-	-	-	-
Mut-miR406	ttctggattaGaaAagcattcta	-	-	-	-
Mut-miR407	tttaccaaaagAaAatgatttaaa	-	-	-	-
Mut-miR408	cGagggaagaggGagtgcat	-	-	-	-
Mut-miR413	tgtgcagaaGaagagaaaGtat	-	-	-	-
Mut-miR414	ttgacgaAgatgaAgaagatga	-	-	-	-
Mut-miR415	atgttctgtAtGtgctctgtt	-	-	-	-
Mut-miR416	tgaacagtgAaGgtacgaacc	-	-	-	-
Mut-miR417	ttcgaacaaaAtGactaccttc	-	-	-	-
Mut-miR418	tggtcagttGaAcatcacatta	-	-	-	-
Mut-miR419	tcaacatcGtGagcattcataa	-	-	-	-
Mut-miR420	ttgcatttcGgAgattagttta	-	-	-	-
Mut-miR426	tcgtaaggaGaaaAttccaaaa	-	-	-	-
Mut-miR447a	caacaaaacaAcAcgtccccaa	-	-	-	-
Mut-miR447c	caacaaaagaAgAcgtccccaa	-	-	-	-
Mut-miR472	gtatgggGggagAaggaaaaa	-	-	-	-
Mut-miR771	agggctacGaGagaggctca	-	-	-	-
Mut-miR773	gagaGaaaagcAggaagcaaa	-	-	-	-
Mut-miR774	gatggccaAaAgggtaaccaa	-	-	-	-
Mut-miR775	ttggcactgGtagaGatcgaa	-	-	-	-
Mut-miR776	tttaacatGaatagaagaGttaga	-	-	-	-
Mut-miR777	agcaacgaaaGtGaatgcgta	-	-	-	-
Mut-miR778	cggtgtacaAaaaGcaagcca	-	-	-	-
Mut-miR779	atgagcagGaaGatagcagaa	-	-	-	-
Mut-miR780,1	acctgctGaacagGtgctaga	-	-	-	-
Mut-miR780,2	atgccagataAtGacgaagaa	-	-	-	-
Mut-miR781	ttttaagtaAcGagaaaactctaa	-	-	-	-
Mut-miR782	aagaacatcGaaggAgtttgt	-	-	-	-
Mut-miR783	gaacatgaaGgagGaaagctt	-	-	-	-
Mut-miR822	atgtgcaaaAgGttcccgca	-	-	-	-
Mut-miR823	tatcttataAgaAcaccaccca	-	-	-	-
Mut-miR824	tcccttctGaGaaatggtcta	-	-	-	-
Mut-miR825	gttcatgcaGcAtcttgagaa	-	-	-	-
Mut-miR826	cacgtatcGaaaaGcggacta	-	-	-	-
Mut-miR827	tagtttgtAgaAggtcatctaa	-	-	-	-
Mut-miR828	tggaatactGaAttaagcaaga	-	-	-	-
Mut-miR829,1	attccatcaAtAggtatcagagct	-	-	-	-
Mut-miR829,2	ttctacctAgaagGtttaatttg	-	-	-	-
Mut-miR830	tttcacttctAcAcaaaatagtta	-	-	-	-

Mut-miR831	caagaagagAaGgaagagatca	-	-	-	-
Mut-miR832-3p	cttgctAggatAgggaatcaa	-	-	-	-
Mut-miR832-5p	tttcgattGcGgatcccagca	-	-	-	-
Mut-miR833-3p	cttgtttgtAgaGatcggtcta	-	1,51887	2,9998	-
Mut-miR833-5p	actagacGgagAacaacaaaca	-	-	-	-
Mut-miR834	ttaccaccgcAaGtgctacca	-	-	-	-
Mut-miR835-3p	ctttcttgcgAaActtctcca	-	-	-	-
Mut-miR835-5p	tttgaAaaagaaGatatgcaagaa	-	-	-	-
Mut-miR836	cgcatGaaaggaaaGacagga	-	-	-	-
Mut-miR837-3p	tccatcagtAtAttgttcgttt	-	-	-	-
Mut-miR837-5p	ttgaaaGgaacaagaaaGtgat	-	-	-	-
Mut-miR838	ttgtgGaagaagAagaagaaaa	-	-	-	-
Mut-miR839	gggaacgaAgaaaggAtggta	-	-	-	-
Mut-miR840	gttagttAaggAccttcagtgt	-	-	-	-
Mut-miR841	ttcagtttGaagAggctcgta	-	-	-	-
Mut-miR842	ggatgacggaAcAgaccatga	-	-	-	-
Mut-miR843	tccaatgaagGtGgacctaaa	-	-	-	-
Mut-miR844	tagcttaAaagGaatcttacca	-	-	-	-
Mut-miR844*	tttaactagAaagaAggcttataa	-	-	-	-
Mut-miR845a	catcaattggAaAcagagccg	-	-	-	-
Mut-miR845b	catcaattAggAatcagagcga	-	-	-	-
Mut-miR846	taattcaagGaGttcaattcaa	-	-	-	-
Mut-miR847	catGaagaagaagaggagAga	-	-	-	-
Mut-miR848	agcttaggGagAcccatgtca	-	-	-	-
Mut-miR849	ttttactacaGcaaAgtttagtta	-	-	-	-
Mut-miR850	ctttgttgAagAccggatctta	-	-	-	-
Mut-miR851-3p	gtcgtctttgAtAgccaccca	-	-	-	-
Mut-miR851-5p	ttgtggatGgGgaaccgaga	-	-	-	-
Mut-miR852	cagaacAaaggGgcttatctt	-	-	-	-
Mut-miR853	cttctcGaagcAaaagagggga	-	-	-	-
Mut-miR854a	ctcctcctcGcAatcctcatc	-1,22757	1,10833	-1,21354	-1,02009
Mut-miR855	ttccttttcGtAagcttttgct	-	-	-	-
Mut-miR856	gctgaagtAaAtggtaggatta	-	-	-	-
Mut-miR857	tttatacaccAtGaacatacaaaa	-	-	-	-
Mut-miR858	aaggtcgaaGagaGaacgaaa	-	-	-	-
Mut-miR859	tttgacttGaGaacagagaga	-	-	-	-
Mut-miR860	tttatacatagAcGaatctattga	-	-	-	-
Mut-miR861-3p	tgtcctAgaagaGatatccatc	-	-	-	-
Mut-miR861-5p	ttgacgcataAtActccaagg	-	-	-	-
Mut-miR862-3p	tcttcaagAagaAccagcatat	-	-	-	-
Mut-miR862-5p	gcacatgctGgaGctattgga	-	-	-	-
Mut-miR863-3p	tattatgtctAgAtgctctcaa	-	-	-	-
Mut-miR863-5p	tttattgagaAcaaGaagacataa	-	-	-	-
Mut-miR864-3p	ttcttcaaggAaAtattgacttta	-	-	-	-
Mut-miR864-5p	ttttgaagtGaaAcatacctga	-	-	-	-
Mut-miR865-3p	tttttggaAaaaAttgaggaaaaa	-	-	-	-

Mut-miR865-5p	tttctcaatAagaAccaaattcat	-	-	-	-
Mut-miR866-3p	tttcttGaaagaGggattttgt	-	-	-	-
Mut-miR866-5p	tttaacaaaaAcGgttccttga	-	-	-	-
Mut-miR867	ttttcctaaAaaaGcatgttcaa	-	-	-	-
Mut-miR868	tgcattatGagGacttaagaag	-	-	-	-
Mut-miR869,1	caacacGagaaAtgaaccaat	-	-	-	-
Mut-miR869,2	gtcaactatGtGaacaccaga	-	-	-	-
Mut-miR870	tgatGgaagaaaGaccaaatta	-	-	-	-
cel-miR-230	tctcctggtGgGacaactaata	-	-1,42075	-	-
cel-miR-231	ctgcctgtAgaAcacgagctt	-5,16643		1,02696	
cel-miR-234	ttaagggtaAtGtcgagcaata				-
cel-miR-234	ttaagggtaAtGtcgagcaata				
cel-miR-235	caggcGggggagagAgcaat				-1,05726
cel-miR-235	caggcGggggagagAgcaat		-		
cel-miR-239a	ccagtacctaAgAgtagtacaa				-
cel-miR-240	gcgaagaAtAgggggccagt				
cel-miR-243	tatcccgcGgGgatcgtacc				
cel-miR-245	gagctactAggaggggaGcaa			-2,5588	
cel-miR-246	gctcctaGcGgaaacatgta			4,96589	
cel-miR-253*	ccttcccaGaaGgcctacta				
cel-miR-254	cctacagtGgGgaaagatttgc				-
cel-miR-255	tttctgAaaaaaaActcttcagtt				
cel-miR-256	tttacagtcAtGtatgcattcc				
cel-miR-258	tttaaaaggaAtGctctcaaaac		-1,26405		
cel-miR-261	ttttcgAgaaaaGtaaaaagct				
cel-miR-262	ttttatGagaaaaGatcgagaaa				
cel-miR-264	tcataacaaGaaGcacccgc				2,04761
cel-miR-265	tataccaccGtAcctccctc	-1,86359	1,03967	-1,76022	-1,12763
cel-miR-265	tataccaccGtAcctccctc	1,23159	-1,01326	1,00216	1,17651
cel-miR-268	accaaactgcAtGtaattcttgc	-			
cel-miR-269	tagttttgcGagagActtgc				3,52727
cel-miR-272	tttcaaacaGcGatgcctac				-
cel-miR-273	tcagccgaGaGagtacgggc				
cel-miR-34	caaccagcAaaGcacactgcc		1,71624		
cel-miR-353	ttaataccaaGaGatggcaatt				-
cel-miR-354	aggagcagGaaGaaacaagg				
cel-miR-356	tgatttgttGgGgttgctca	5,0962			
cel-miR-358	gaccttgaGagggaAaccaa				
cel-miR-36	catgcgaatAtAcacccggtg				-
cel-miR-37	ctgcaagtgAtGacccggtg		-1,26221		
cel-miR-39	caagctgattAaGacccggtg				
cel-miR-41	taggtgattAtAcacccggtg			7,648	
cel-miR-42	tctgtagaAgAtaacccggtg				-
cel-miR-43	gcgacagGaagAaaactgtgat				-
cel-miR-44	ttagctgaaAgAgtctctagtc				
cel-miR-56	ctcagGggaaaGattacgggt				-

cel-miR-57	acacagctGgaActacagggt		-1,80943		
cel-miR-58	attgccgtaGtgaaGgatctc	26,80462	-	-	-
cel-miR-58	attgccgtaGtgaaGgatctc	9,19667	24,13219	-	-
cel-miR-59	catcatccAgaAaaacgattcg			2,35331	
cel-miR-60	ttgaacAagaaaaAgtgcataat		-8,30569		
cel-miR-61	ttgatgagAaaGggttctagtc			7,03533	
cel-miR-64	tcggtaacgGtAcagtgtcat		1,51921		
cel-miR-64	tcggtaacgGtAcagtgtcat			4,89952	
cel-miR-71	tttttcactaGcGatgtctttc				-
cel-miR-73	ctgaactgccAaGatcttgcc				
cel-miR-76	tcaaggcttGaAcaacaacga				8,6711
cel-miR-77	tggacagcAaAggcctgatga		-		
cel-miR-78	gcacaaaGaaGcaggcctcc	1,42114			
cel-miR-784	tctacgtaGgGagattgtgcc		-	-	-
cel-miR-785	ttctacaGaaaaGaattcactt	-	-	3,93563	-
cel-miR-79	agctttggAaaGctagcttta	-	-	-	-
cel-miR-790	gcggtgttGgGgagtgccaa	-	-	-	-
cel-miR-793	tttctgtcAaacAaagatacctc	-	-	-	-
cel-miR-793	tttctgtcAaacAaagatacctc	-	-	-	-
cel-miR-797	tcttctcatAgAgattgctgtgat	-	-	-	-
cel-miR-799	ccactagcttAaAcagggttc	-	-	-	-
cel-miR-800	cggcagacaaAtAccgagttt	-7,96548	-	-	-
cel-miR-81	actagctttGaGgatgatctc	-	-	-	-
dme-bantam	aatcagctAtGaaaatgatctc	-	-	-	-
dme-let-7	ttttactataGaaGctactacctc	-	-2,00743	-5,29869	-
dme-miR-11	gcaagaaGtGagactgtgat	-2,01628	-2,01145	-4,19345	2,58788
dme-miR-13b	actcgtGaaaaAggctgtgat	-	-	-	-
dme-miR-184*	gcccttatGagAtctccgtcc	-	-	-	-1,12655
dme-miR-219	caagaattgGgAttggacaatc	-	-	-	-
dme-miR-263b	gtgaattcAcGcagtgccaa	-	-	-	-
dme-miR-276*	cgtaggaacAcAatacctcgct	-	-	-	-
dme-miR-280	tcatttcataAgGaacgtaaatac	-	-	-	-
dme-miR-281-2*	actgtcgaGggaAagctctct	-	-	-	-
dme-miR-282	gacaaagccAagAagaggctagat	-	-	-	-
dme-miR-286	gcacgagtgAtGggtctagtc	-	-	-	-
dme-miR-286	gcacgagtgAtGggtctagtc	-9,22185	-	-	-
dme-miR-289	gtcgcaggctcGaGttaaatattt	-	-	-	-
dme-miR-2b	ctcctGaaagcAggctgtgat	-	-	-1,20546	-
dme-miR-303	accagtttccAgAgaaacctaa	-	-	-	-
dme-miR-305	cagagcaccAgaAgaagtacaa	-	-	-	-
dme-miR-308	tctcacagAaAaatcctgtgat	-	-	-	-
dme-miR-309	taggacaaaGtAtacccagtg	-	-	-	-
dme-miR-310	aaggcGgggaagAgtgcaat	-	-	-1,10203	-
dme-miR-311	caggccggAgaaAgtgcaat	-	-	-47,75176	-
dme-miR-318	tgagataaaGaaagGccagtg	-	-	-	-1,44691
dme-miR-33	caatgcgacAaGaatgcacc	-12,8998	-	-	-

dme-miR-33	caatgcgacAaGaatgcacc	1,1695	1,36876	-1,33301	4,07635
dme-miR-4	tttcaatggtAgActagcttta	-	-1,40784	-	53,29779
dme-miR-87	tcacaccAgaaaAtttgctca	-	-	-	-
dme-miR-87	tcacaccAgaaaAtttgctca	-2,11126	-6,8763	-2,69832	-1,42477
dme-miR-92a	taggcGgggaGaagtgcaat	-	-	-	-
dme-miR-9b	catacagcAaaaaAcaccaaag	-	2,23145	-	-

Table S2. ***cis*-elements identified in the *MIR168a* and *MIR168b* promoters**. The copy number of each element in each promoter is indicated.

	cis-element	Sequence	miR168a	miR168b	Function feature
**Pathogens, Elicitors, Salicylic acid**	**ERE (Elicitor Responsive Element)**	**TTGACC**	1	2	Elicitor Responsive Element core of parsley PR1 genes; binding site of WRKY1 and WRKY2 TFs
	**WRKY 71 binging site**	**TGAC**	11	14	Binding site of rice WRKY71 and parsley WRKY TFs
	**W-box NPR1**	**TTGAC**	5	4	W-box found in promoter of Arabidopsis thaliana NPR1 gene; recognized specifically by SA-induced WRKY TFs
	**SEBF PR-10a**	**TTGTCAC**<br>**TTGTCTC**	2<br>1	1	Binding site of the potato silencing element binding factor (SEBF) gene found in promoter of pathogenesis-related gene (PR-10a)
	**GT-1**	**GAAAAA**	6	7	GT-1 motif found in the promoter of the soybean SCaM-4. Plays a role in pathogen and salt-induced SCaM-4 gene expression
	**G-box**	**CACGTG**	6	4	Tomato Pti4 (ERF TF) regulates gene expression via GCC box (GTTAGTT, Myb1) and non-GCC box (CACGTG, G-box) cis-elements
	**ASF-1 binding site**	**TGACG**	0	2	cis element activation sequence-1 (as-1)-like elements are recognized by ASF-1 and the TGA family of TFs; involved in transcriptional activation of several genes by auxin and salicylic acid (i.e. *AtPR1a*)
**Auxin**	**Auxin response factor (ARF) binding site**	**TGTCTC**	1	1	ARF (auxin response factor) binding site found in the promoters of primary/early auxin response genes of *Arabidopsis thaliana*
	**NDE (SAUR)**	**CATATG**	2	2	Sequence found in NDE element in soybean SAUR (Small Auxin-Up RNA) 15A gene promoter
	**NtBBF1 binding site**	**ACTTTA**	3	4	NtBBF1 (Dof protein) binding site in Agrobacterium rhizogenes. Required fro tissue-specific expression and auxin induction
	**SURE (Sulfur-responsive element)**	**GAGAC**	3	5	Core of sulfur-responsive element (SURE) found in the promoter of the Arabidopsis SULTR1 gene; contained in the ARF binding sequence (GAGACA)
**Jasmonic Acid**	**T/G-box**	**AACGTG**	1	1	T/G-box found in tomato proteinase inhibitor II (pin2) and leucine aminopeptidase (LAP) genes
**Ethylene**	**LeCp binding site**	**TAAAATAT**	2	0	Core element in the promoter of tomato LeAcs2 gene (Cys protease) required for fungal elicitor Ethylene-Inducing Xilanase (EIX) responsiveness (ethylene biosynthesis)
**Gibberelin**	**GARE**	**TAACAGA**	1	1	Gibberellin-responsive element (GARE) found in the promoter region of a cysteine proteinase gene in rice
	**Pyrimidine box**	**CCTTTT**	2	2	Gibberellin-response cis-element of GARE and pyrimidine box are partially involved in sugar repression
	**CARE**	**CAACTC**	1	1	CAREs (CAACTC) found in the promoter region of a cystein proteinase gene in rice

Table S3. Sequences of oligonucleotides used in this study. Listed are the primers used for qRT-PCR analysis of pre-miR168 and *AGO1* expression, oligonucleotides used as probes for Northern blot analyses, and primers used for preparation of plant transformation vectors.

Oligo ID	Accession number	Sequence
**Expression analysis by qRT-PCR**		
Pre-miR168a	At4g19395	Forward : 5’-ATTCGCTTGGTGCAGGTC-3’
		Reverse : 5’-ACCTCGGACTCCGATTCAGTT-3’
pre-miR168b	At5g45307	Forward: 5’-GATTCGCTTGGTGCAGGT-3’
		Reverse : 5’-TCGAGGATCCGATTCAGTTGAT-3’
AGO1	At1g48410	Forward : 5'-CAAGATGCACACGCTCAGTT-3'
		Reverse : 5'-TCTACCAGCCATTCCACCTC-3'
FLC1	At5g10140	Forward: 5'-CTGGCCGATTCTCACTTGAT-3'
		Reverse: 5'-CAATTACAAGGATGGACCTA-3'
Ubiquitin-10	At5g65080	Forward: 5'-ACACTTCACTTGGTCTTGCGT-3'
		Reverse: 5'-AGTCTTTCCGGTGAGAGTCTTCA-3'
**Stem-loop qRT-PCR**		
miR168		ST-primer: 5’-GTTGGCTCTGGTGCAGGGTCCGAGGTATTCGCACCAG AGCCAACTTCCCG-3’
		Reverse: 5’- GGCGGTCGCTTGGTGCAGGT-3’
**Probes for northern blot analysis**		
miR171-5p	At3g51375	5'-GATATTGGCGCGGCTCAATCA-3'
siRNA1003		5'-ATGCCAAGTTTGGCCTCACGGTCT-3'
miR415	MI0001426	5'-ATGTTCTGTATGTGCTCTGTT-3'
U6	At3g14735	5'-TCATCCTTGCGCAGGGGCCA-3'
**Plant Transformation vectors**		
miR168a sensor	At4g19395	Forward : 5'-GGATCCATGAAGACTAATCTTTTTC-3'
		Reverse : 5'- GGATCCTAGCTTGATGCAGCTCGGGAAAATTTAAAGCT CATCATGTTTGTATAGT-3'
miR168a promoter	At4g19396	Forward : 5'-GGTACCTGAGTGGAGTTAGTTGTA-3'
		Reverse : 5'- AAGCTTAGACTCTACTATCATAATAATCCC -3'
