# Supplementary material for: Small RNA profiling reveals regulation of Arabidopsis miR168 and heterochromatic siRNA415 in response to fungal elicitors
Source: BMC Genomics. 2014 Dec 10;15(1):1083. doi: 10.1186/1471-2164-15-1083 (PMC4299684; doi:10.1186/1471-2164-15-1083)
Supplement: Supplementary file 2 — Additional file 2: Figure S1: Frequency of occurrence of defence-related cis-elements in the MIR168a promoter generated in POBO (http://ekhidna.biocenter.helsinki.fi/poxo/pobo/pobo). Figure S2. Accumulation of siRNA415 in control (c) and elicitor-treated (e) Arabidopsis plants. (PDF 222 KB) [file 12864_2014_6774_MOESM2_ESM.pdf]

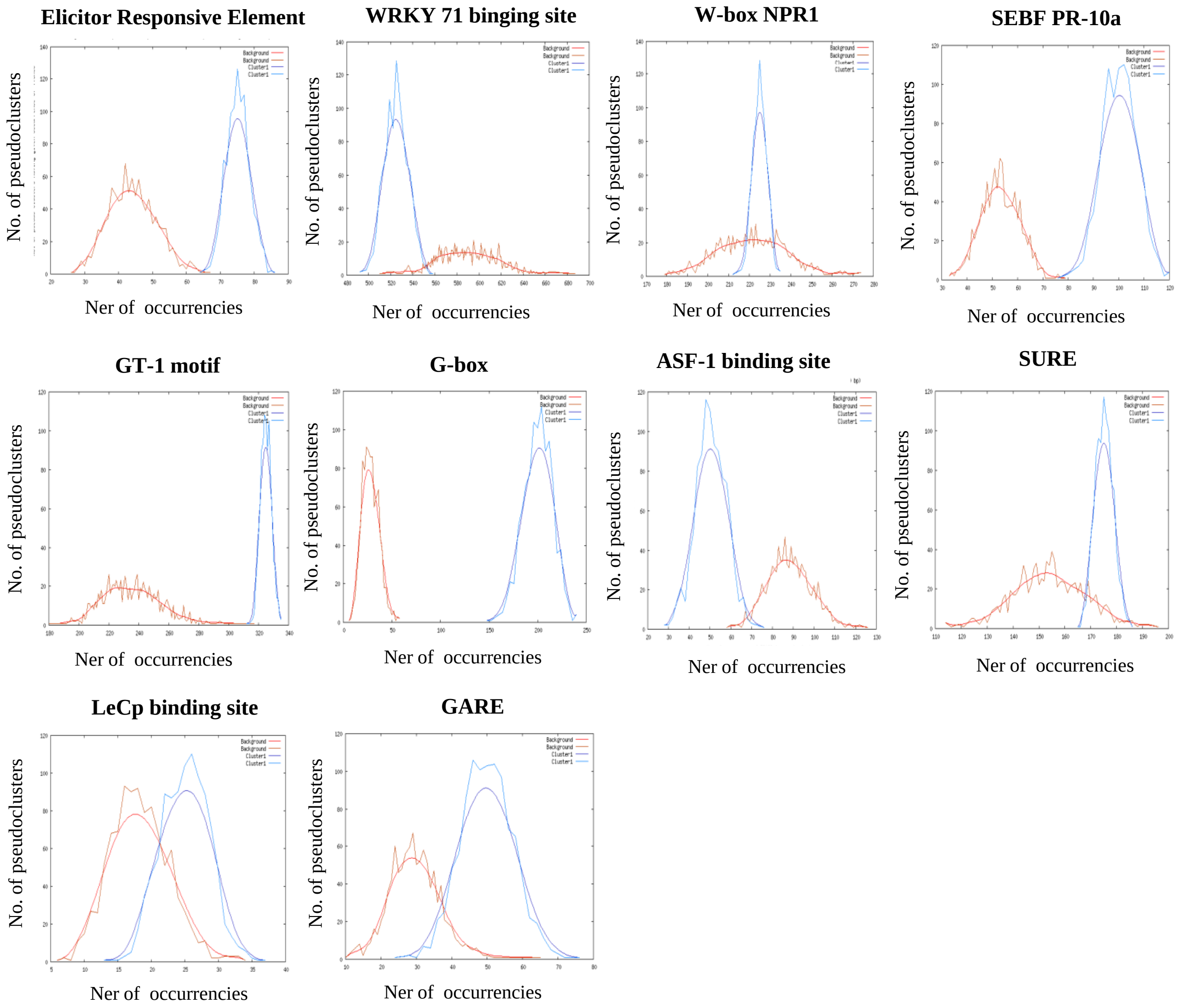

Figure S1. **Frequency of occurrence of defence-related *cis*-elements in the *MIR168a* promoter generated in POBO for Arabidopsis thaliana background promoters**. The 2kb sequences upstream of the transcription start site were analysed in POBO application (http://ekhidna.biocenter.helsinki.fi/poxo/pobo/pobo) (Kankainen and Holm, 2004), to determine the frequency of occurrence of each motif. Except for the W-boxNPR1 motif, all the other motifs were found to be significantly over-represented in the *MIR168a* promoter (p-value < 0.0001).

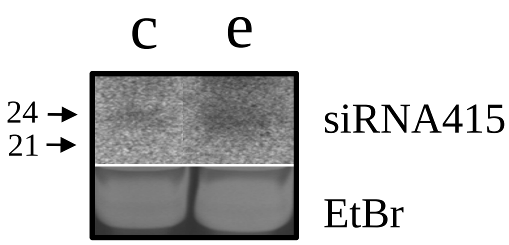

Figure S2. **Accumulation of siRNA415 in control (c) and elicitor-treated (e) Arabidopsis plants**. Elicitor treatment was carried out for 30min. This small RNA sequence has been annotated in miRBase as miR415. Results obtained in this work, however, demonstrated that this smRNA is not a miRNA but a hc-siRNA. Accordingly, we re-named this smallRNA as siRNA415. Ethidium bromide-stained rRNAs are shown as loading controls.
